# Synthesis of Antimicrobial Benzimidazole–Pyrazole Compounds and Their Biological Activities

**DOI:** 10.3390/antibiotics10081002

**Published:** 2021-08-19

**Authors:** Maria Marinescu

**Affiliations:** Department of Organic Chemistry, Biochemistry and Catalysis, Faculty of Chemistry, University of Bucharest, Soseaua Panduri, 030018 Bucharest, Romania; maria.marinescu@chimie.unibuc.ro

**Keywords:** benzimidazole, pyrazole, hybrids, antimicrobial, pharmaceutical properties

## Abstract

The synthesis of new compounds with antimicrobial and antiviral properties is a central objective today in the context of the COVID-19 pandemic. Benzimidazole and pyrazole compounds have remarkable biological properties, such as antimicrobial, antiviral, antitumor, analgesic, anti-inflammatory, anti-Alzheimer’s, antiulcer, antidiabetic. Moreover, recent literature mentions the syntheses and antimicrobial properties of some benzimidazole–pyrazole hybrids, as well as other biological properties thereof. In this review, we aim to review the methods of synthesis of these hybrids, the antimicrobial activities of the compounds, their correlation with various groups present on the molecule, as well as their pharmaceutical properties.

## 1. Introduction

Microbial resistance is one of the burning issues facing clinical practice and finding new effective compounds against multi-resistant pathogens is one of the major goals in current biomedical research [1]. The discovery of new antimicrobial and antiviral compounds is a major goal in the context of today’s COVID-19 pandemic [2]. It is known that both patients with severe cases and patients with moderate cases of COVID-19, with or without pneumonia, have received treatments with various antibiotics [3]. Among the heterocyclic compounds known in the literature for their antimicrobial activities are those with benzimidazole ring and those with pyrazole ring, aromatic compounds with a very wide range of medicinal properties. Benzimidazole derivatives developed a considerable interest in medical domain due to their therapeutic action as antitumor [4,5,6,7], antimicrobial [8,9,10,11,12,13,14], antihelmintic [15], antihistaminic [16,17], proton pump inhibitors [16,18], anti-inflammatory [19,20] and anti-hypertensive [21] drugs. Astemizole-related compounds demonstrated anti-prion activity for the treatment of Creutzfeldt–Jakob disease, while albendazole compounds are currently used as medication for the treatment of a variety of parasitic worm infestations. Additionally, benzimidazoles treat mitochondrial dysfunction in Alzheimer’s disease [22], possess neurotropic, psychoactive, analgesic effects [23], anticoagulant proprieties [24] and are efficient agents in Diabetes mellitus [25]. Additionally, pyrazole compounds possess a diversity of biological activities as analgesic [26,27,28], anticonvulsivant [29,30], antitumor [31,32,33,34], antidiabetic [35,36], antimicrobial [37,38,39,40,41,42,43], antipyretic [44,45], antiviral [46,47], antimalarial [48,49], local anesthetic [50] and so forth.

Moreover, the literature mentions a series of benzimidazole–pyrazole hybrids with remarkable antimicrobial properties, and not only, antiviral activities, even anti-COVID-19 [51,52,53,54], in the context of the new pandemic, which has led us to current research, to study their synthesis methods, antimicrobial properties, structure–property relationships, and their biological activities.

**Figure 1:**
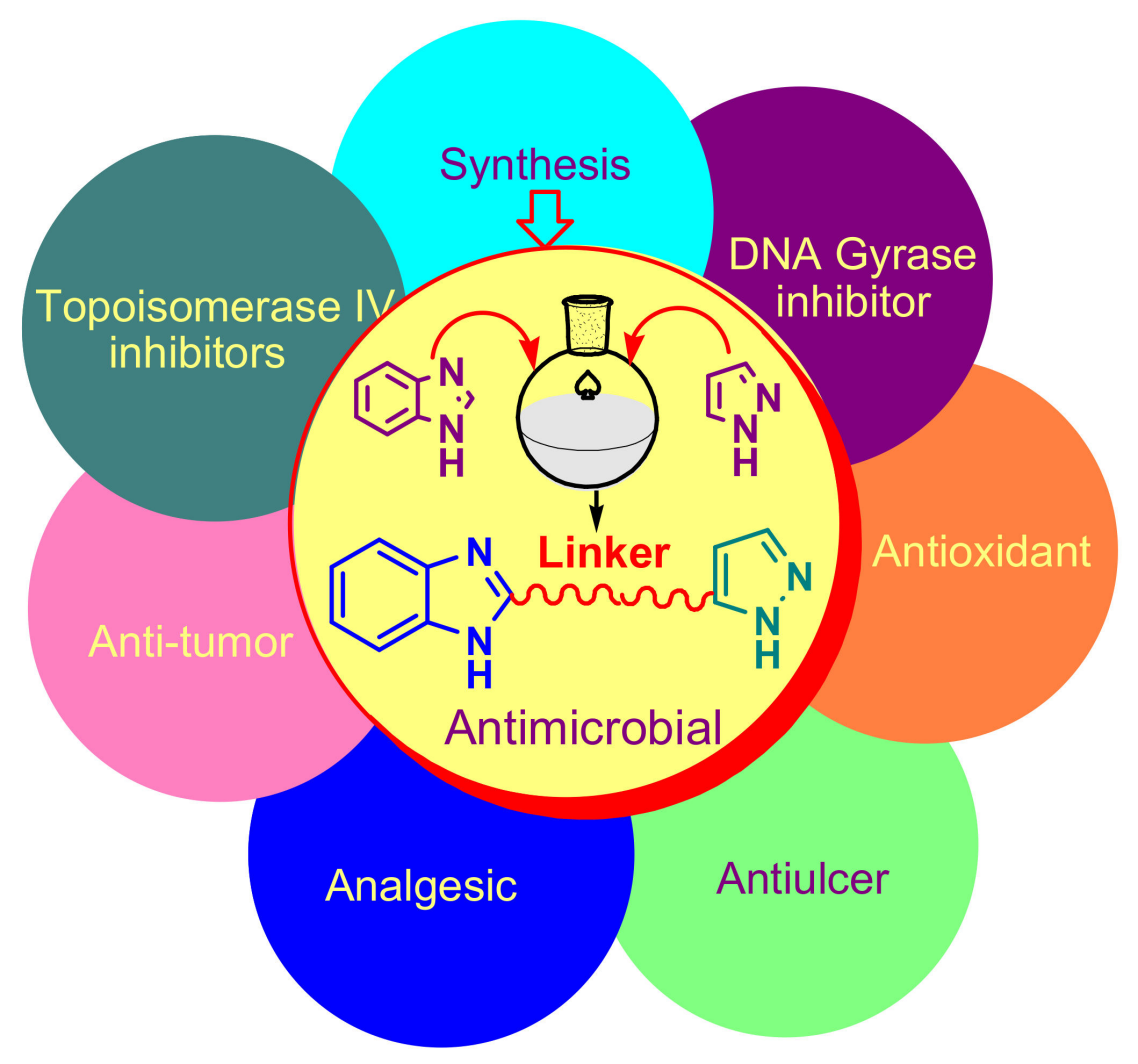
Schematic representation of the synthesis and biological properties of benzoimidazole–pyrazole compounds.

In this review, we aim to review the various methods of synthesis of benzimidazole–pyrazole hybrid compounds with antibacterial and antifungal properties, DNA-Gyrase inhibitors, topoisomerase IV inhibitors, as well as the other biological properties they possess, such as: antitumor, antioxidant, anti-inflammatory, analgesic, antiulcer (Figure 1). In order to highlight the structures of the heterocycles in the discussed compounds, we colored the benzimidazole nucleus with blue, the pyrazole with green, the linker with red, and the compounds with good biological activity are marked with a rectangle.

## 2. Synthesis, Antimicrobial Activities of Benzimidazole–Pyrazole Compounds. Benzimidazole–Pyrazole Compounds as Potent DNA Gyrase and Topoisomerase IV Inhibitors

### 2.1. Benzimidazoles Substituted in the “2” Position with Pyrazole Moiety

**Scheme 1:**
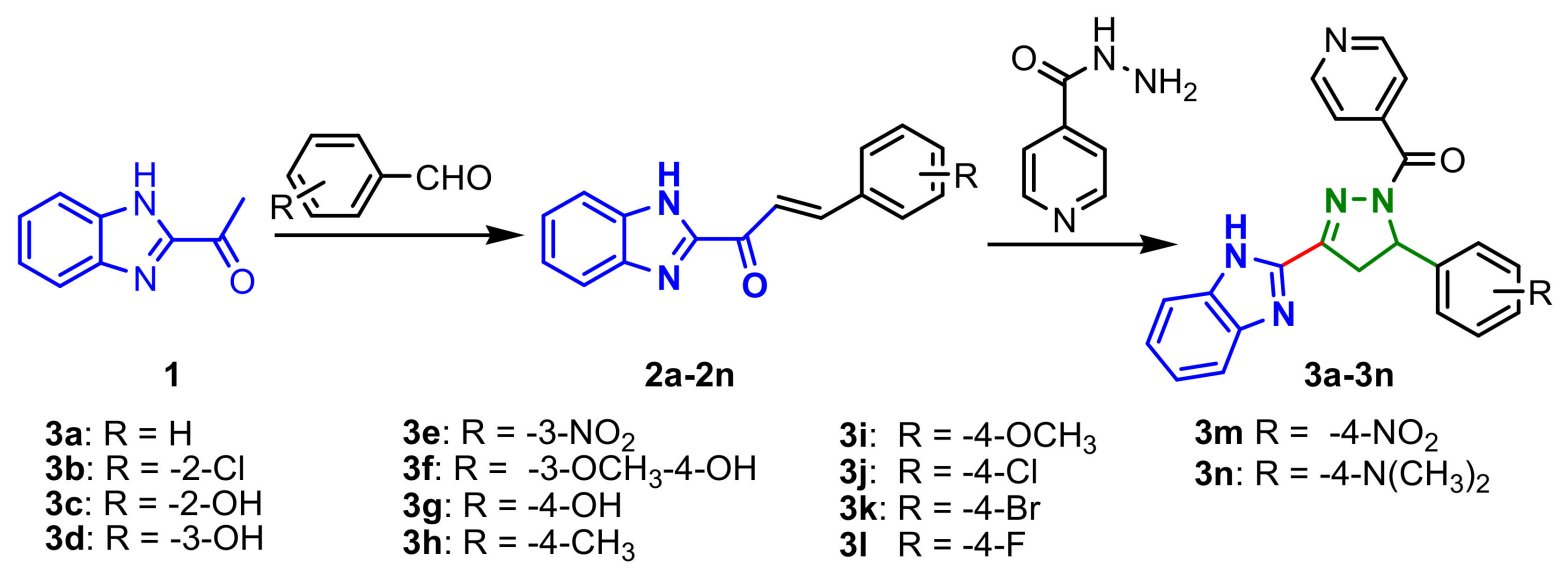
Synthesis of benzimidazole–pyrazoles **3a**–**3n**.

Benzimidazole chalcones **2a**–**2n**, synthesized from 2-acetylbenzimidazole **1** and aldehydes in ethanolic KOH by a Claisen–Schmidt condensation, were cyclocondensated with izoniazide, to give (3-(1*H*-benzo[d]imidazol-2-yl)-5-(aryl)-4,5-dihydro-1*H*-pyrazol-1-yl)(pyridin-4-yl)methanones **3a**–**3n** in good yields (Scheme 1). All compounds showed antimicrobial activity against bacterial strains *E. coli*, *P. aeruginosa*, *S. aureus*, *S. pyogenes* and fungi *C. albicans*, *A. niger* and *A. clavatus*. The compounds **3d**, **3g**, **3h**, were found to be the best antibacterials, with MIC of 25 μg mL^−1^ against *P. aeuginosa* (**3d**) and *E. coli* (**3g**, **3h**) and compound **3n** the best antifungal, with MIC 25 μg mL^−1^ against *A. niger* [55]. 

**Scheme 2:**
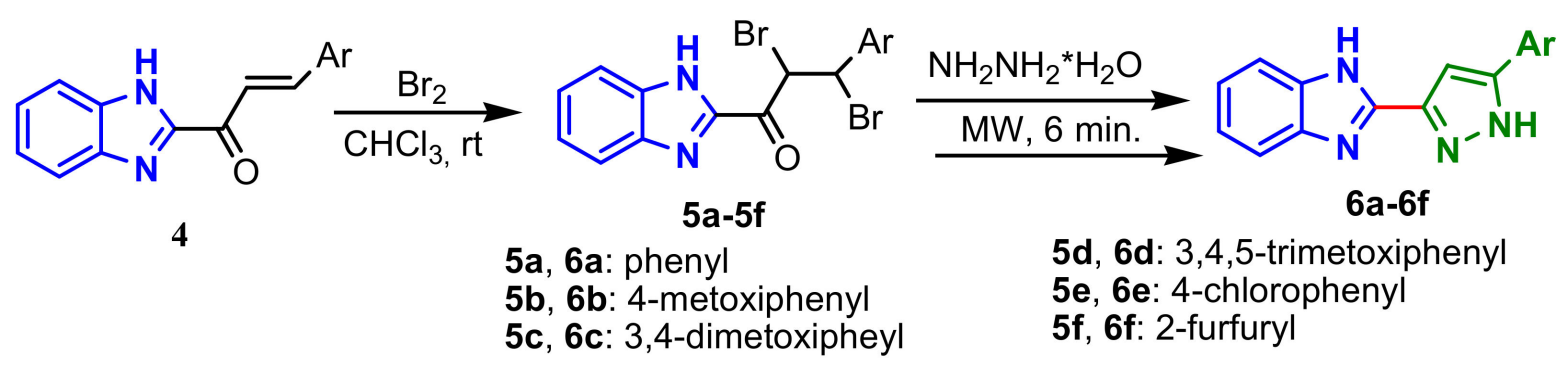
Synthesis of benzimidazole-pyrazole **7**.

Rajora and Srivastava reported the synthesis of some 2-(1*H*-pyrazol-3-yl)-1*H*-benzo[d]imidazoles by bromination of benzimidazolyl chalcone **4**, with the formation of dibrominated intermediates **5a**–**5f**, followed by cyclization in the presence of hydrazine hydrate and dehydrobromination, with the formation of compounds **6a**–**6f** (Scheme 2). 

Compounds **6a**–**6f** showed good antimicrobial activity on four bacterial strains, *E. coli*, *P. aeruginusa*, *B. subtilis*, *K. pneumoniae* and two fungi, *Candida albicons* and *Aspergillus niger*, considering ciprofoxacin and fluconazole as standard drugs [56]. 

**Scheme 3:**
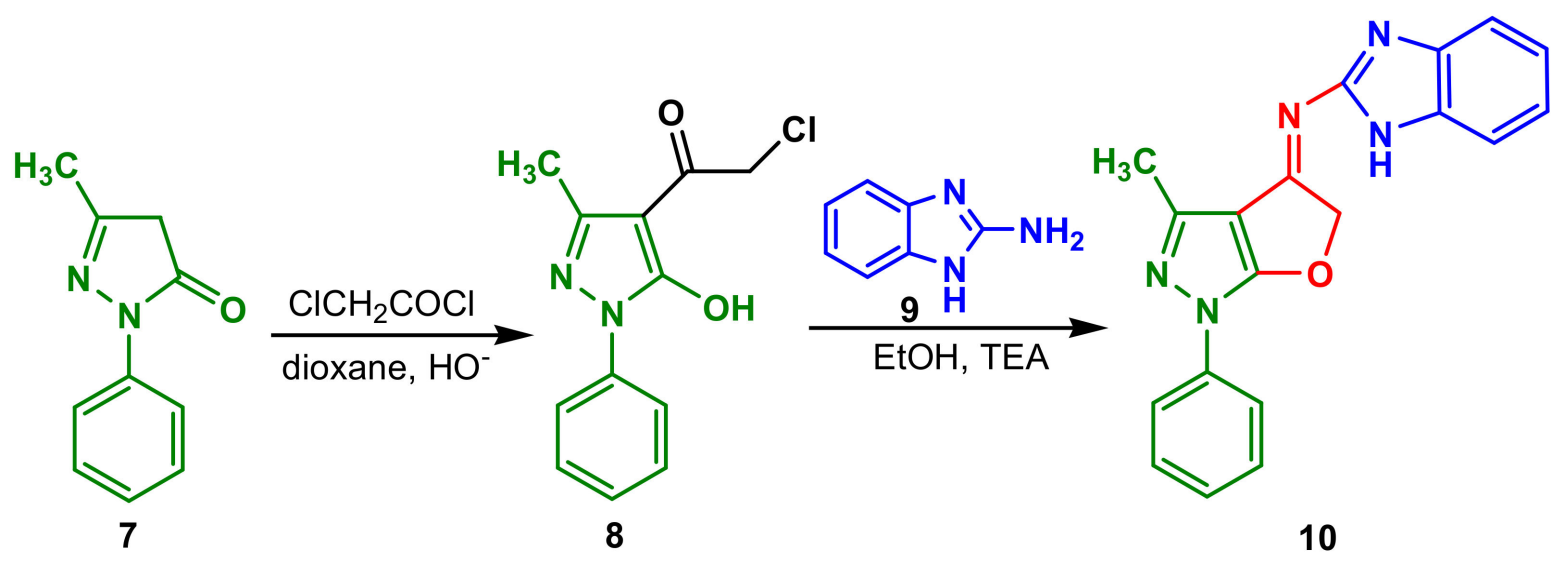
Synthesis of benzimidazole–pyrazole **10**.

2-Chloro-1-(5-hydroxy-3-methyl-1-phenyl-1*H*-pyrazol-4-yl)ethanone **8** synthesized by refluxing 5-pyrazolone **7** with chloroacetyl chloride in a basic dioxane solution reacted with 2-aminobenzimidazole **9** to give N-(3-methyl-1-phenyl-1*H*-furo [2,3-c]pyrazol-4(5*H*)-ylidene)-1*H*-benzimidazol-2-amine **10** (Scheme 3) [57]. Compound **10** showed a very good anti-Gram-positive profile, being equivalent to chloramphenicol against *B. subtilis* (MIC 3.125 μg mL^−1^), significant activity against *B. thuringiensis* (MIC 6.25 μg mL^−1^) and also good antibacterial activities against Gram-positive bacteria, *E. coli* (MIC 50 μg mL^−1^) and *P. aeruginosa* (MIC 50 μg mL^−1^). Antifungal activity of the compound **10** was 50% lower than cycloheximide in inhibitory the growth of *B. fabae* and *F. oxysporum* (MIC 6.25 μg mL^−1^).

**Scheme 4:**
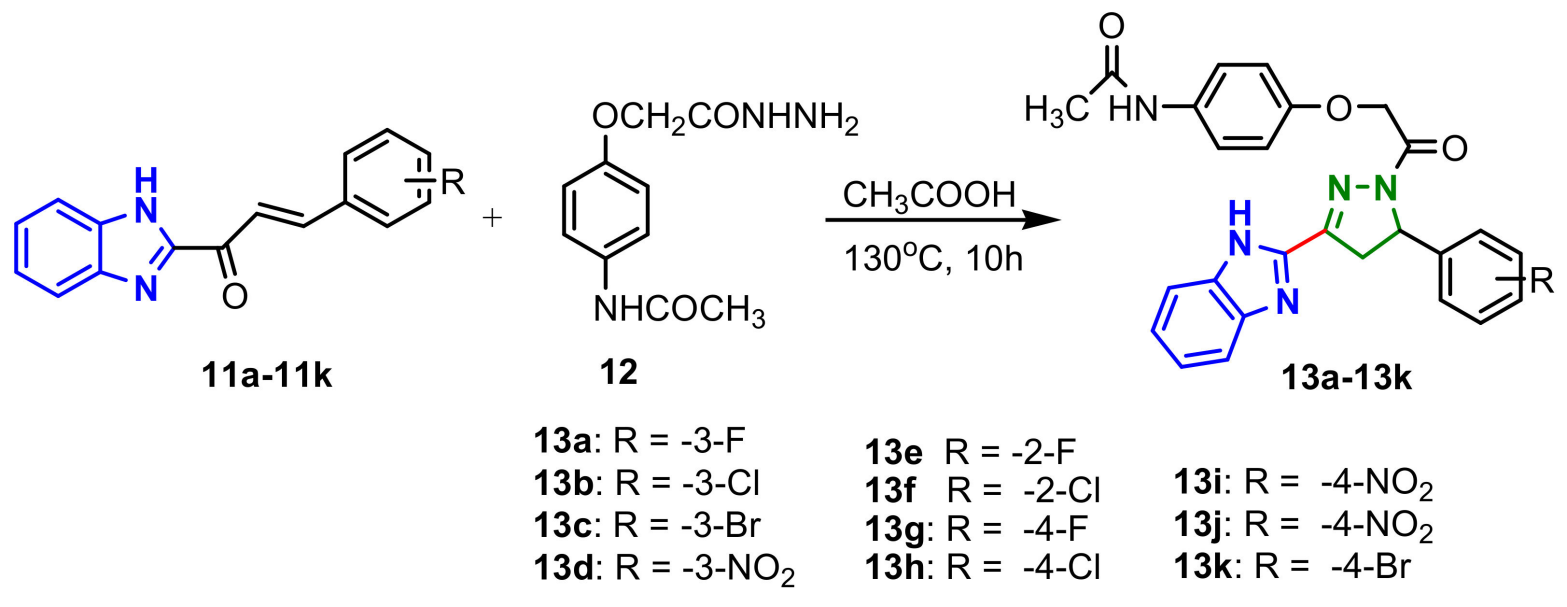
Synthesis of benzimidazole–pyrazoles **13a**–**13k**.

A similar condensation of chalcones **11a**–**11k** with intermediate hydrazide **12** in acetic acid, at 130 °C afforded new benzimidazole bearing pyrazoline derivatives **13a**–**13k** in excellent yields (Scheme 4). All compounds showed antimicrobial activity against bacteria *E. coli* MTCC443, *P. aeruginosa* MTCC1688, *S. aureus* MTCC 96, *S. pyogenes* MTCC 442 and fungi *C. albicans* MTCC 227, *A. niger* MTCC 282 and *A. clavatus* MTCC 1323. Compounds **13a**–**13d** showed the highest inhibition against almost all bacteria tested with values of minimum inhibitory concentrations of 25–50 mg mL^−1^, while derivatives **13e**–**13k** had antifungal activity against almost all strains tested, with similar CMI values [58]. Structure–activity relationship studies have shown that the presence of electron-withdrawing groups in the aromatic ring, like F, Cl, Br and NO_2_, are responsible for increasing antimicrobial activity for most microorganisms tested.

**Scheme 5:**
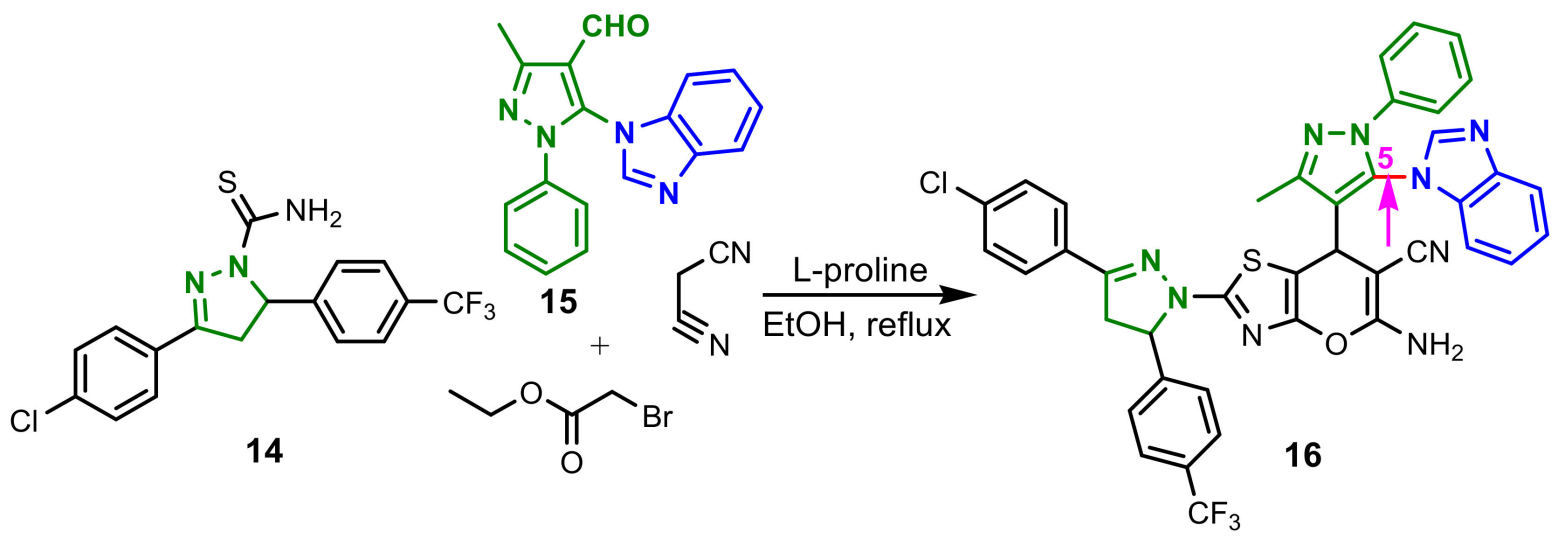
Synthesis of benzimidazole–pyrazoles **16**.

Kalaria et al., reported a L-proline promoted one-pot four-component tandem reaction for synthesis of compound **16**, starting from carbothioamide **14**, pyrazolyl aldehyde **15**, α-bromoethylacetate and malononitrile (Scheme 5) [59]. Antibacterial activity of the compounds **16** was screened against three Gram-positive bacteria (*Streptococcus pneumoniae* MTCC 1936, *Bacillus subtilis* MTCC 441 and *Clostridium tetani* MTCC 449) and three Gram-negative bacteria (*Escherichia coli* MTCC 443, *Salmonella typhi* MTCC 98, *Vibrio cholerae* MTCC 3906) using ampicillin, norfloxacin and ciprofloxacin as the standard antibacterial drugs. Compound **16** illustrated an excellent activity against Gram-positive bacteria *B. subtilis* (62.5 μg mL^−1^), being more potent than ampicillin (250 μg mL^−1^) and norfloxacin (100 μg mL^−1^) and also against *C. tetani*, with a CMI of 200 μg mL^−1^ compared with 250 μg mL^−1^ for ampicilin. Additionally, the structure–activity relationship (SAR) showed that the presence of benzimidazole in the fifth position in the pyrazole ring is responsible for its biological activity. 

**Scheme 6:**
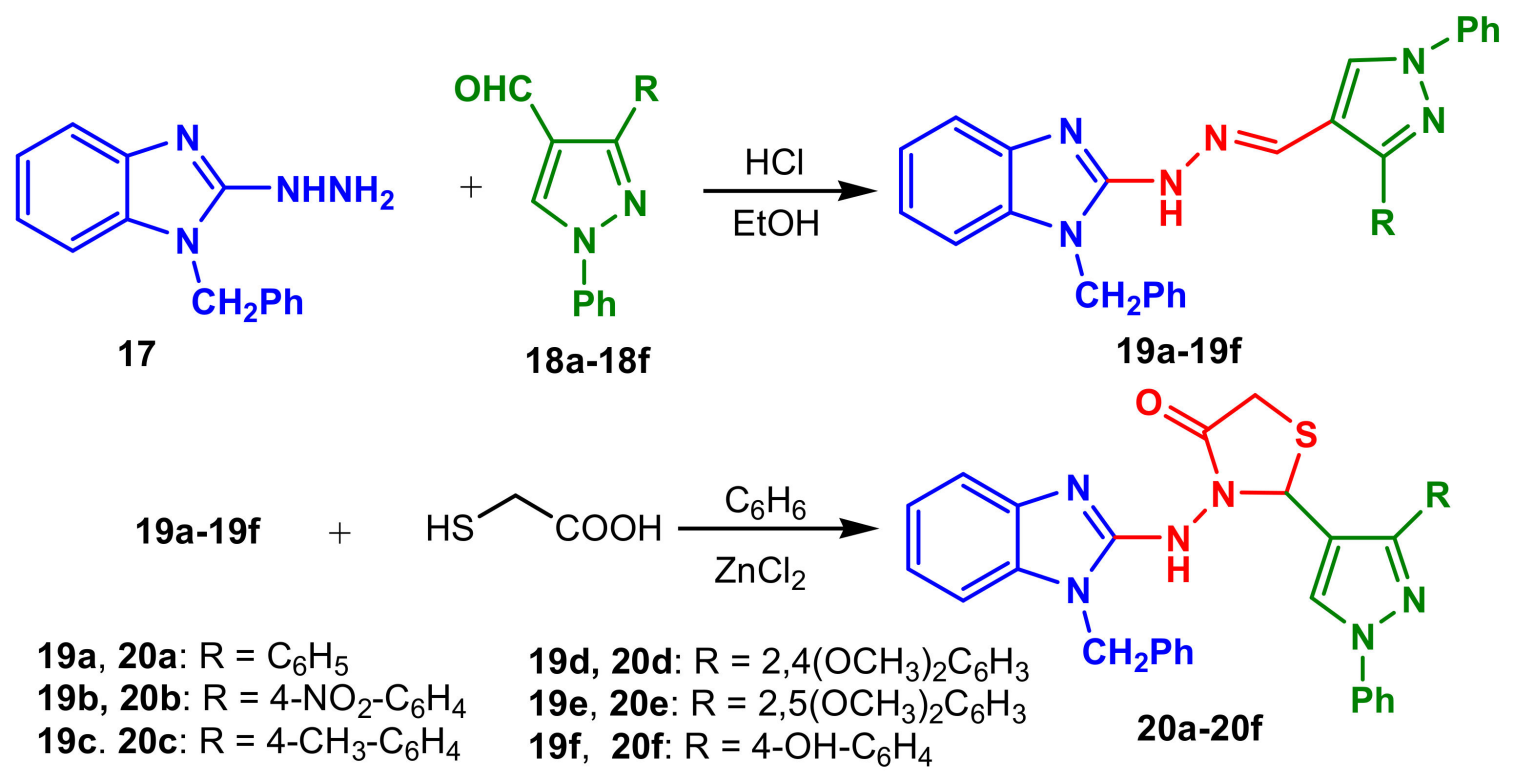
Synthesis of benzimidazole–pyrazoles **19a**–**19f** and **20a**–**20f**.

Patil et al., reported two series o benzimidazole–pyrazole compounds **19a**–**19f** and **20a**–**20f** in two steps: a condensation between 2-benzimidazolehydrazine **17** and pyrazole **18a**–**18f**, followed by cyclization with thioglicolic acid (Scheme 6) [60]. The compounds **19b**, **19d**, **20a** and **20f** show good activity against bacteria *P. aeruginosa*, *S. aureus* and *P. vulgaris*, while the others show moderate to poor activity against all pathogens. The compounds **19a** and **19c** exhibited good activity against fungal strains *A. niger* and *A. flavus.*

**Scheme 7:**
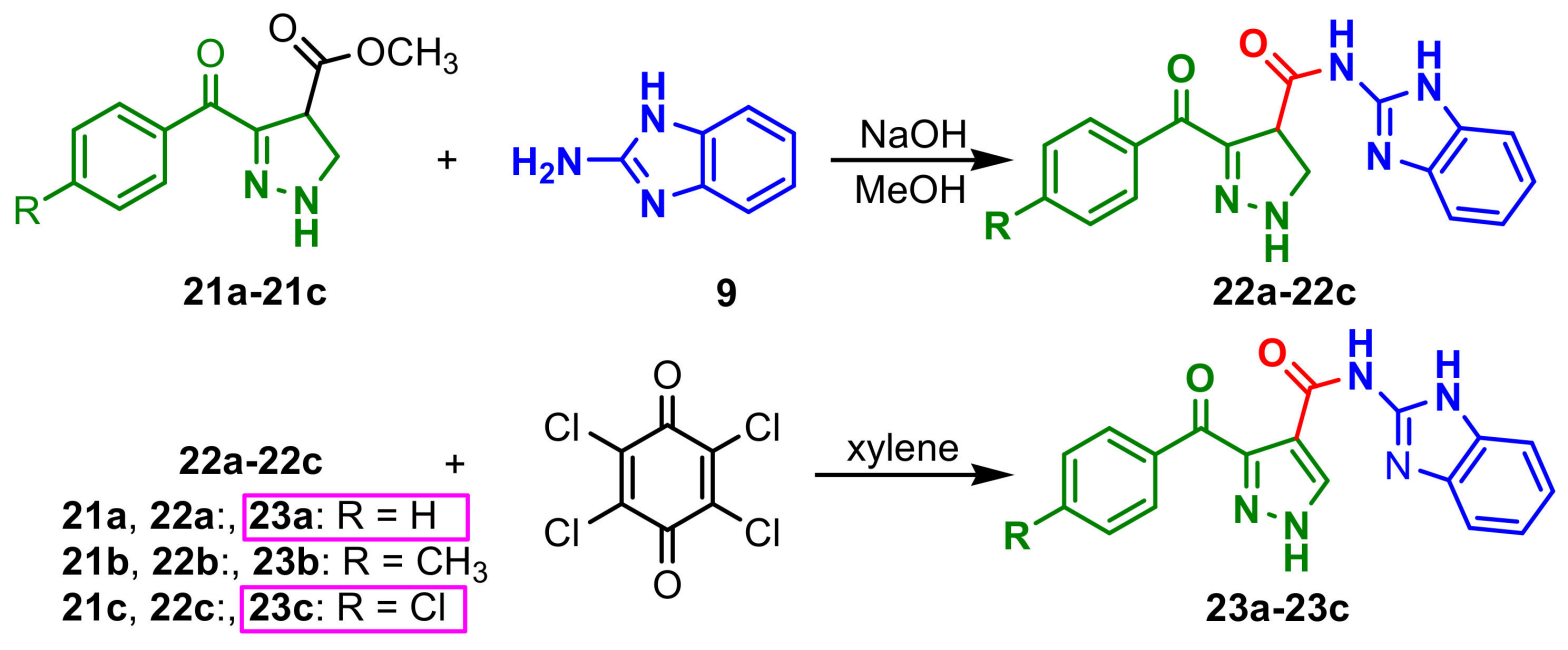
Synthesis of benzimidazole–pyrazoles **23a**–**23c**.

Reddy et al., reported the synthesis of a new class of pyrazolyl–benzimidazoles **23a**–**23c** possessing an amide group by reaction between pyrazolones **21a**–**21c** with 1*H*-benzo[d]imidazol-2-amine **9**, and the oxidation of the intermediate compounds **22a**–**22c** with chloranil (Scheme 7) [61]. It was found that the presence of electron-withdrawing substituent “Cl” on the aromatic ring increases the antimicrobial activity, compound **23c** being a potent antifungal agent against *A. niger* considering ketoconazole as standard. Additionally, compounds **23a** and **23c** possess antimicrobial activity against *B. subtilis* and *P. aeruginosa* (chloramphenicol standard). 

**Scheme 8:**
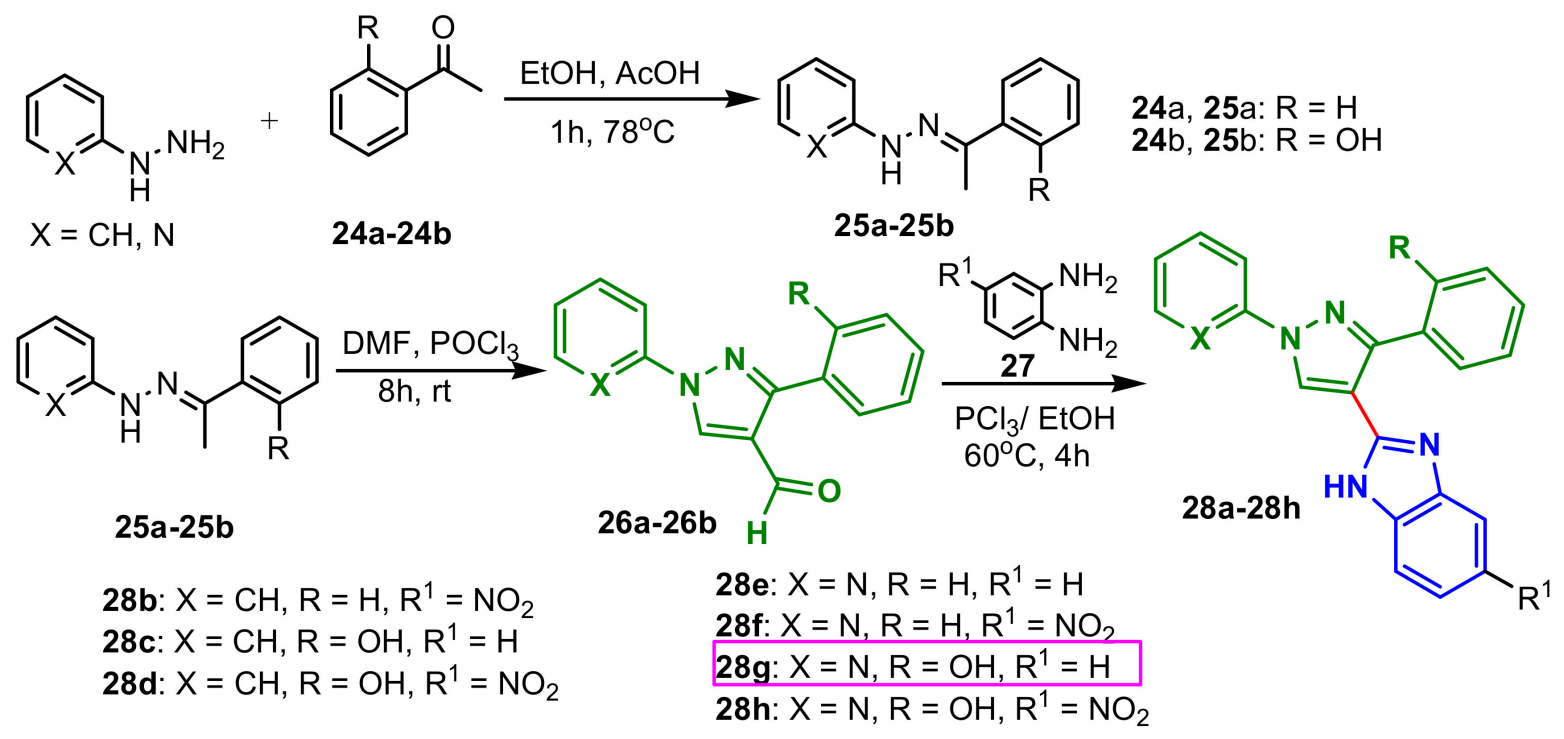
Synthesis of benzimidazole–pyrazoles **27a**–**27i**.

**Scheme 9:**
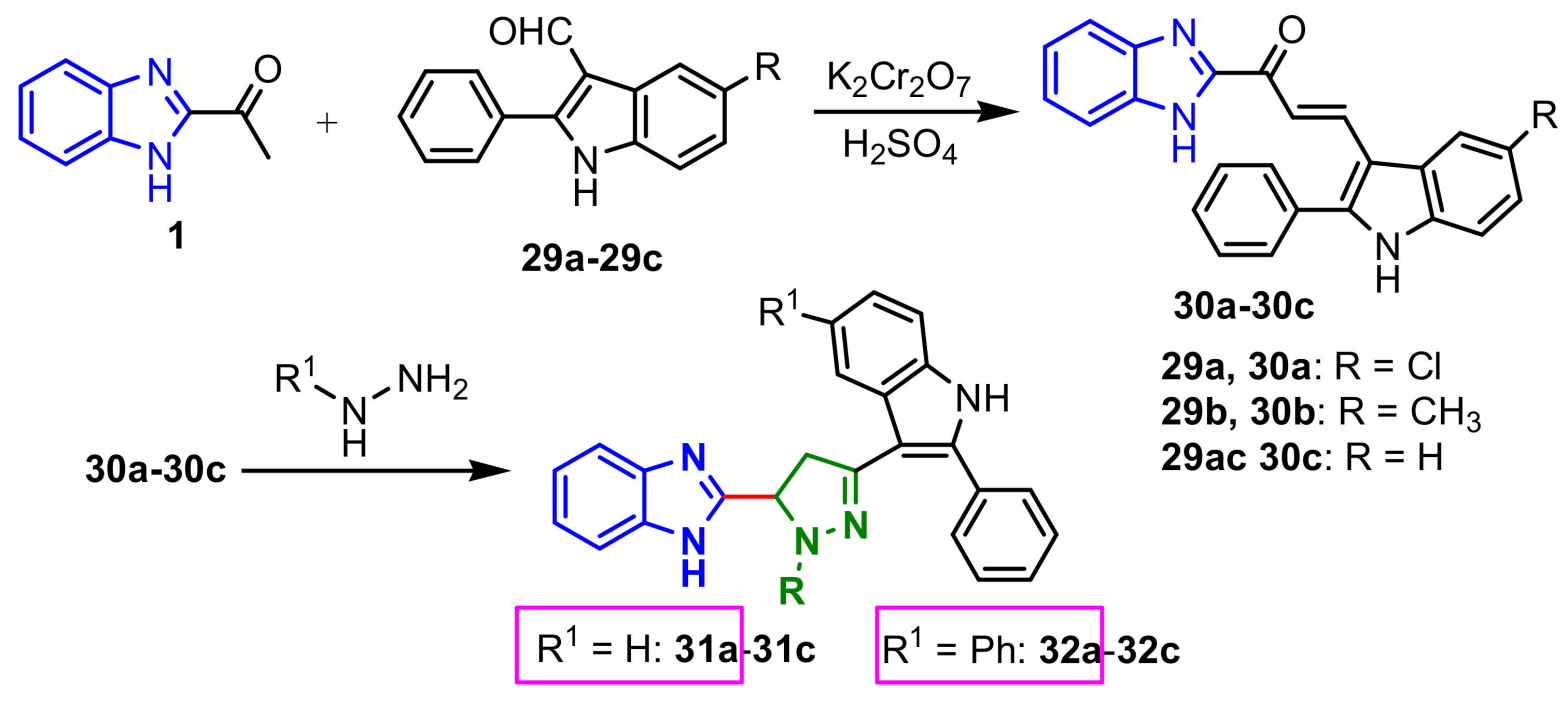
Synthesis of benzimidazole–pyrazoles **31a**–**31c** and **32a**–**32c**.

Padalkar et al., synthesized a new class of antimicrobial agents, by reaction of phenyl hydrazine with substituted acetophenones **24** to give the corresponding hydrazones **25**, which on Vilsmeier–Haack reaction with POCl_3_–DMF gave substituted 3-aryl-4-formyl pyrazoles **26**. Compounds **25a**–**25b** were condensed with o-substituted aromatic amines **27** in the presence of PCl_3_ in ethanol to obtain corresponding 2-[substituted-1*H*-pyrazol-4-yl]-1*H*-benzimidazoles **28b**–**28i** (Scheme 8) [62]. The compound **28g** showed good antibacterial activity against *Escherichia coli* and *Staphylococcus aureus*, and compounds **28d**, **28e**, **28h** exhibited weak to moderate growth inhibitory activity against both *E. coli* and *S. aureus* as revealed from their MIC values (Table 1). Compounds **28f** and **28h** show good inhibitory growth in the case of *Candida albicans* (MIC = 62.5 μg mL^−1^). Saundane et al., reported the synthesis of a series of benzimidazole–pyrazole compounds using a two-step strategy (Scheme 9): synthesis of intermediate chalcones **25a**–**25b** by a condensation reaction, followed by a cyclization reaction with hydazine (compounds **31**) or phenylhydrazine (compounds **32**) [63]. All compounds were assessed for their in vitro antibacterial activity against four representative bacterial species *E. coli* (MTCC-723), *S. aureus* (ATCC-29513), *K. pneumonia* (NCTC-13368) and *P. aeruginosa* (MTCC-1688) using gentamycin as a reference and for their antifungal activity against *A. oryzae* (MTCC-3567T), *A. niger* (MTCC-281), *A. flavus* (MTCC-1973), *A. terreus* (MTCC-1782). Compounds **31a** and **32a** possess good antibacterial and antifungal activity (Table 2), against *E. coli*, *S. aureus* (MIC = 8 μg mL^−1^) and *A. niger* (MIC = 8 μg mL^−1^ for **31a**). Additionally, all compounds possess antioxidant activity.

**Scheme 10:**
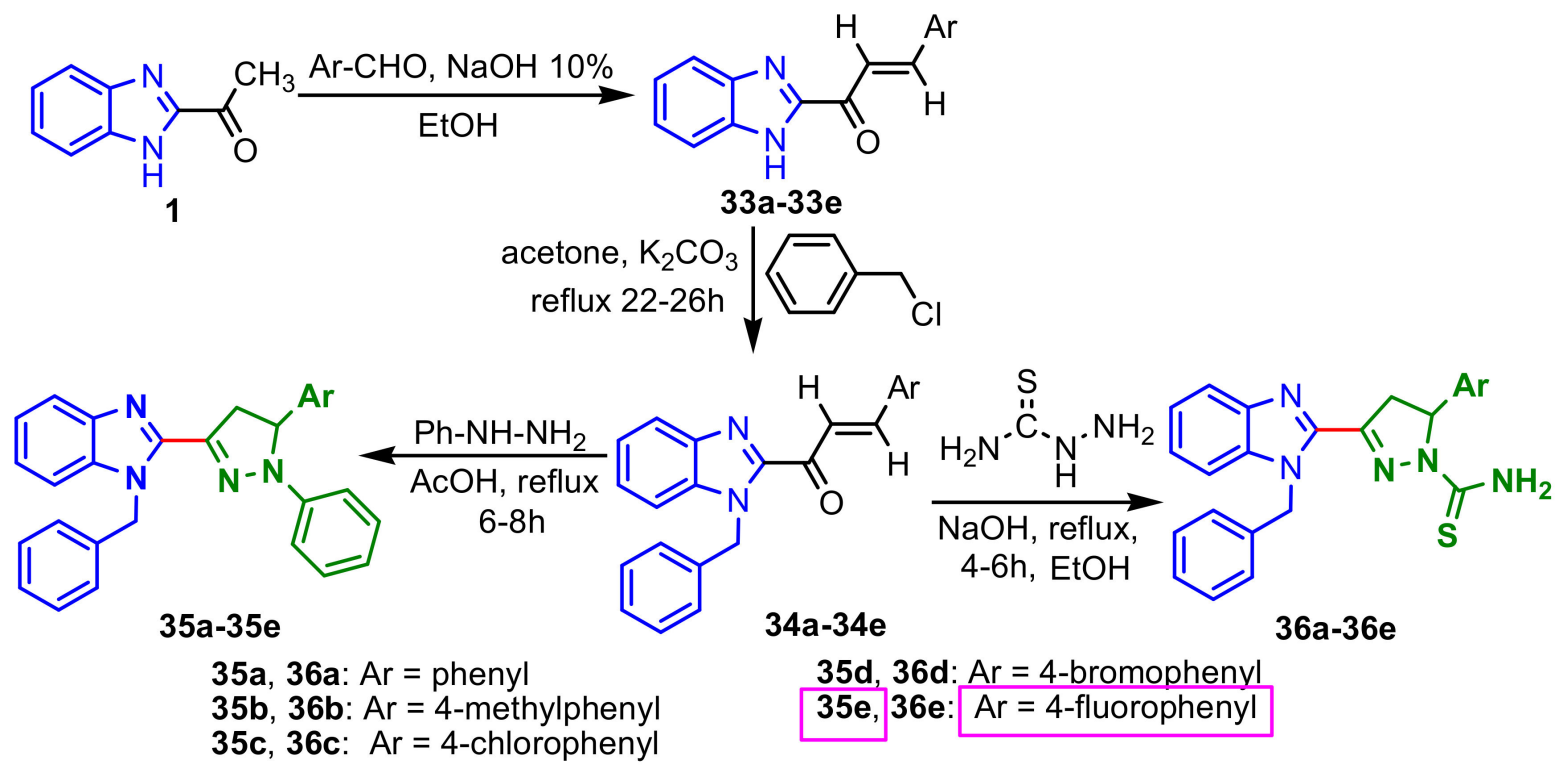
Synthesis of benzimidazole–pyrazoles **35a**–**35e** and **36a**–**36e**.

Padhy et al., synthesized two series of benzimidazole–pyrazole compounds in three steps: (i) Claisen–Schmidt condensation of 2-acetylbezimidazole **1** with substituted aromatic aldehydes in presence of NaOH, to give the intermediates chalcones **33a**–**33e**; (ii) condensation of the chalcones **33** with benzyl chloride gave the corresponding 1-benzyl substituted compounds **34a**–**34e**; (iii) the reaction of compounds **34** with phenylhydrazine in the presence of acetic acid afforded 1-benzyl-2-(5-aryl-1-phenyl-4,5-dihydro-1*H*-pyrazol-3-yl)-1*H*-benzimidazoles **35a**–**35e**, while (iv) condensation with thiosemicarbazide in presence of NaOH, give 5-aryl-3-(1-benzyl-1*H*-benzimidazol-2-yl)-4,5-dihydro-1*H*-pyrazole-1-carbothioamides **36a**–**36e** in good yields (Scheme 10). The in vitro antimicrobial activity of compounds **35**–**36** was tested against four bacterial strains, *S. aureus*, *B. subtilis*, *E. coli*, *P. aeruginos*a, and one fungus, *C. albicans*. The compounds exhibited weaker antimicrobial activities compared to those of the control drugs (Ciprofloxacin and Fluconazole), the MIC values of the compounds ranged between 64–1024 μg mL^–1^ for the 1-phenylpyrazolines **35a**–**35e** and between 128–512 μg mL^–1^ for the pyrazoline-1-carbothioamides **36a**–**36e**. Compound **35e** showed good activity (64 μg mL^–1^) against all tested bacterial strains [64]. 

**Scheme 11:**
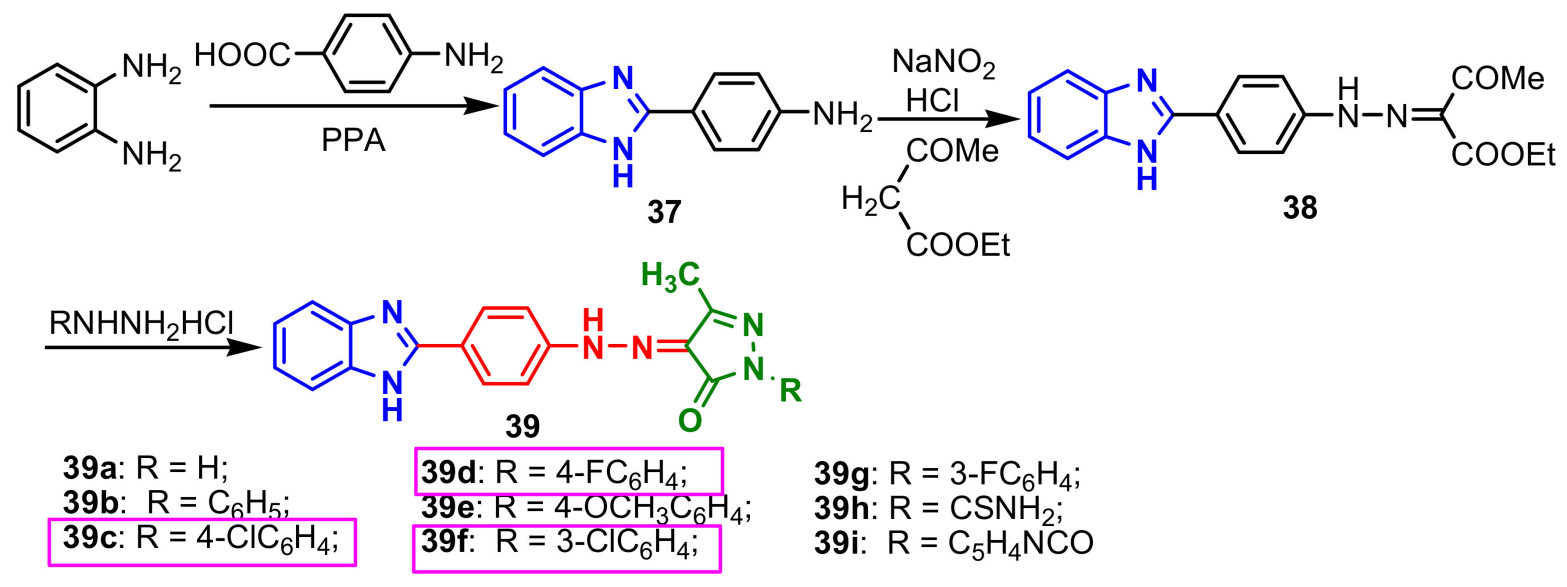
Synthesis of benzimidazole–pyrazoles **39a**–**39i**.

4-(1*H-*benzimidazol-2-yl)benzenamine **37**, obtained by cyclization reaction of 1,2-phenylenediamine with 4-amino benzoic acid, was diazotized and treated with ethylacetoacetate to produce ethyl 2-(2-(4-(1*H*-benzimidazol-2-yl)phenyl) hydrazono)-3-oxobutanoate **38** through intramolecular rearrangement reaction. Dehydrative cyclisation of **38** in the with different hydrazine hydrochlorides produce corresponding benzimidazole–pyrazole **39a**–**39i** (Scheme 11) [65]. The antitubercular and antimicrobial activity of compounds **39** was determined on four Gram-positive strains, three Gram-negative strains and 2 fungi. In Table 3 we marked in green the very good antimicrobial activities of compounds **39c** and **39f**, as well as of the compounds **3d** and **3g**, for the labeled strains, and the very good antibacterial activities of all compounds against *Staphylococcus aureus*. The values of the minimum inhibitory concentrations (MIC) in Table 3 showed that compounds, **39c** and **39f**, possess almost all MICs as good as the standards used for antitubercular and antimicrobial activities, and their antifungal activities are twice as high, compared to Ketoconazole, ie 3.9 μg mL^–1^ against *Aspergillus niger* ATCC 9029 and 1.95 μg mL^–1^ against *Aspergillus fumigatus* ATCC 46645. With the exception of compounds **39f**, **39h** and **39i**, all compounds showed better antibacterial activity (MIC = 125 μg mL^–1^) than standard, Ciprofloxacin, compounds **39c** and **39d** being 16 times more active(MIC = 7.81 μg mL^–1^) than standard.

**Scheme 12:**
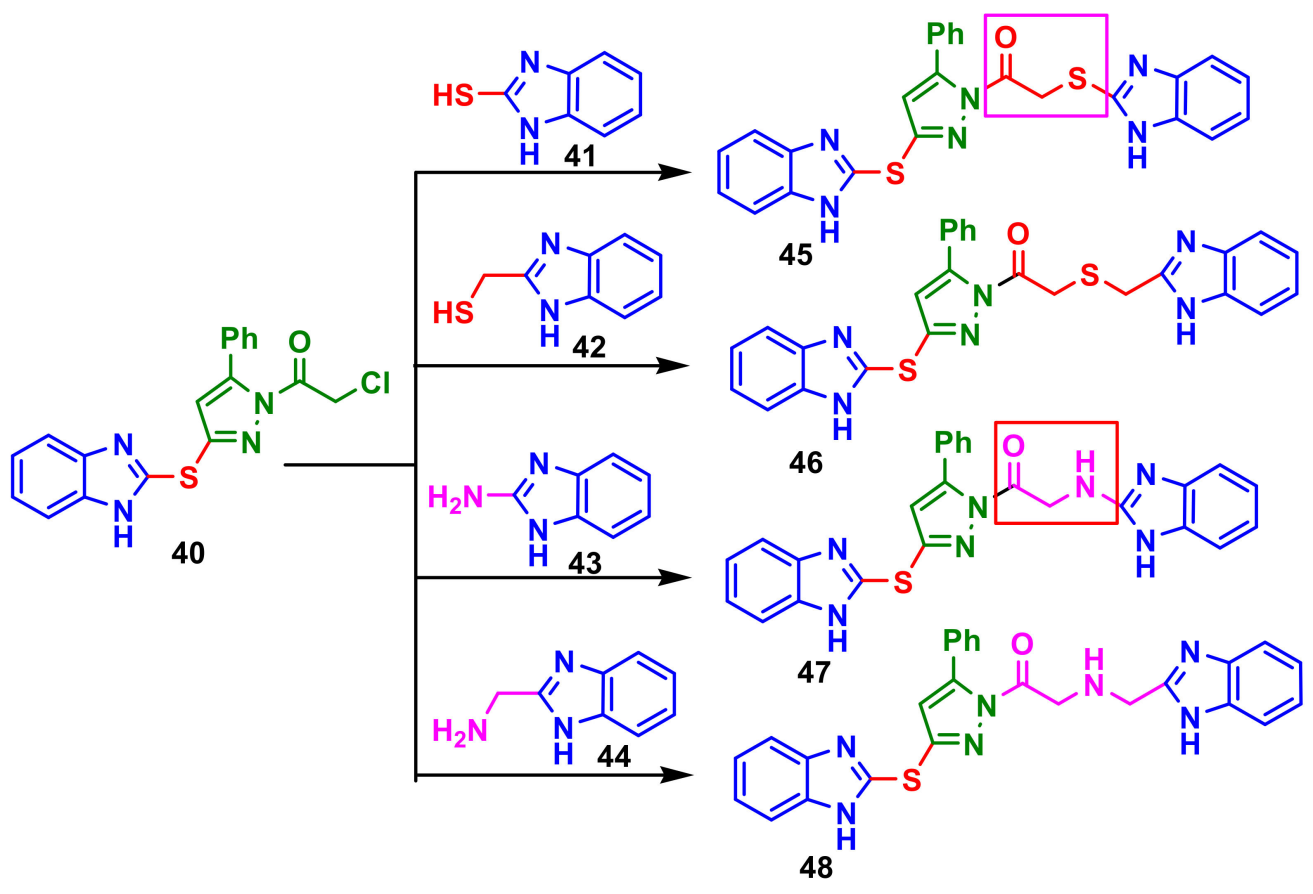
Synthesis of dibenzimidazole–pyrazoles **45**–**48**.

Suram et al., reported the synthesis of a series of bis(benzimidazolyl)pyrazole compounds from chlororacetylpyrazole-benzimidazole **40** and benzimidazoles **41**–**44**, to obtain compounds **45**–**48** (Scheme 12) [66]. It was observed that the compound with *thio ethanone* linkage **45** and *amino ethanone* linkage **47** displayed slightly higher activity than that with methyl thio ethanone **46** and methyl amino ethanone linkage **48** on the microbial tested strains, *S. aureus*, *B. subtilis*, *P. aeruginosa*, *K. pneumoniae*, *A. niger* and *P. chrysogenum*, when compared with the standard drugs chloramphenicol and ketoconazole. 

**Scheme 13:**
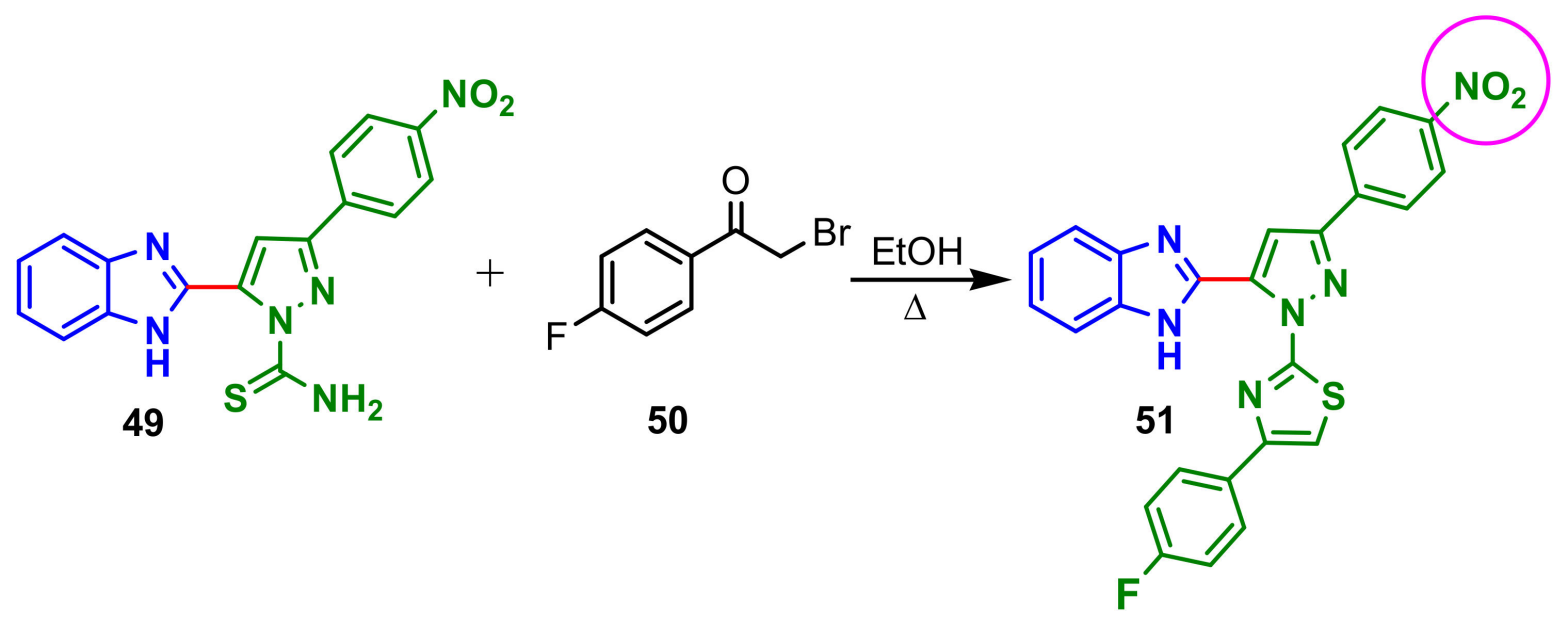
Synthesis of dibenzimidazole–pyrazole **51**.

A new class of benzimidazole–pyrazoles was prepared using a Claisen–Schmidt reaction [67]. From all synthesized compounds, derivative **51**, obtained by cyclocondensation reaction of thioamide **49** with 4-fluorophenacyl bromide **50** (Scheme 13), having nitro substituent on the aromatic ring showed greater antimicrobial activity particularly against *Pseudomonas aeruginosa*, with an inhibition zone of 34 mm at 100 μg per well, and *Penicillium chrysogenum*, with an inhibition zone of 41 mm at 100 μg per well.

**Scheme 14:**
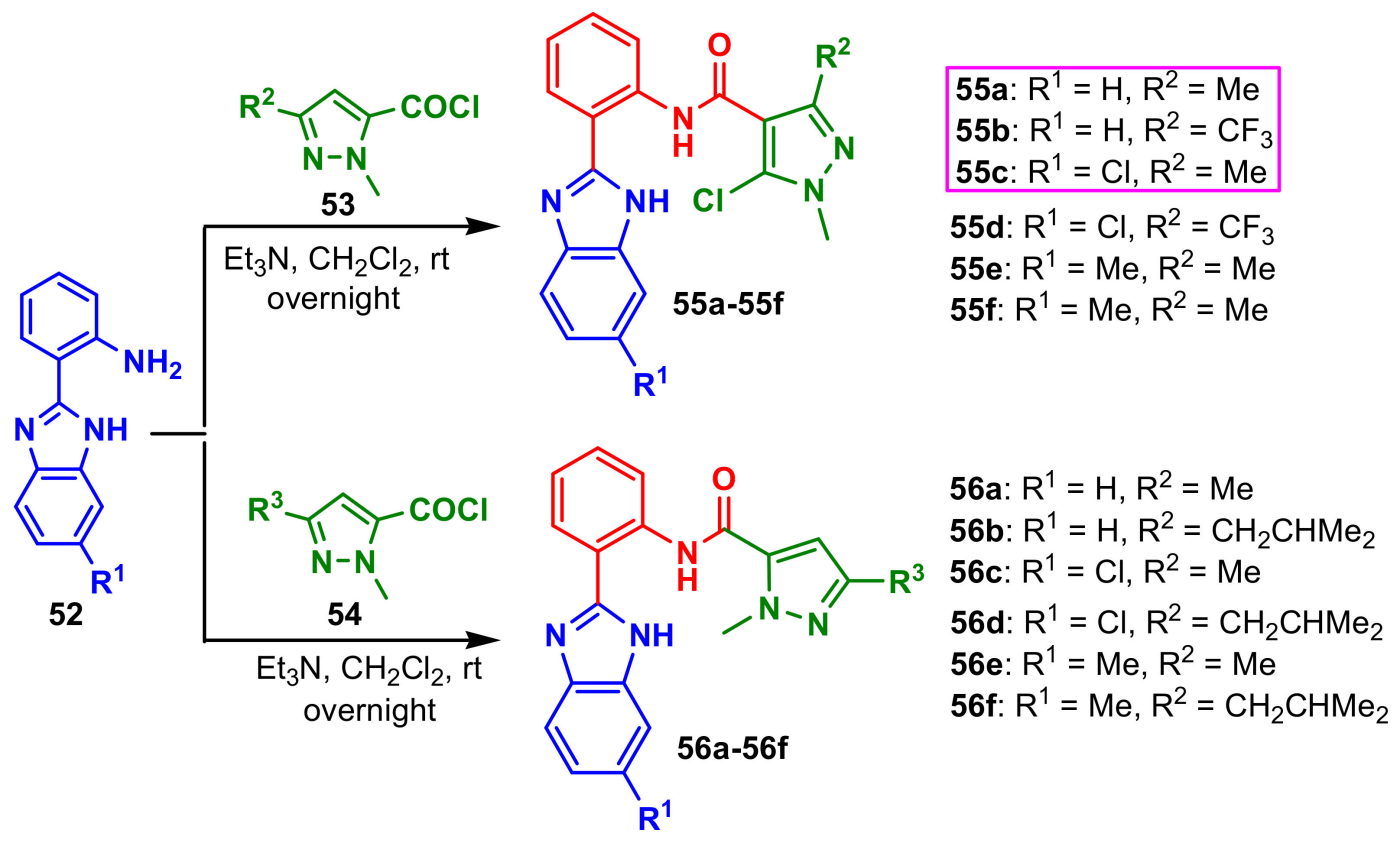
Synthesis of dibenzimidazole–pyrazole **55a**–**55f** and **56a**–**56f**.

Si et al., synthesized two series of benzimidazole–pyrazoles by reaction of benzimidazole **52** with pyrazole-5-carbonyl chlorides **53** and **54** to afford the final compounds **55a**–**55f** and **56a**–**56f** (Scheme 14) [68]. The authors reported the antifungal activities against four fungi, *B. cinerea*, *R. solani*, *F. graminearum*, *A. solani*, considering hymexazol as the positive control at 100 μg mL ^−1^ (Table 4). All compounds showed better inhibitory activity against *B. cinerea*. The inhibition rates of compounds **55a**–**55f** exceeded 60% against *R. solani* and the inhibition rates of compounds **55a**–**55f** ranged from 58.28% to 68.28% against *A. solani*, which were better than 55.43% of the control hymexazol. The compounds with pyrazole-4-carboxamide moiety **55a**–**55f** showed higher activities than the target compounds with pyrazole-5-carboxamide moiety **56a**–**56f**. Thus, the activities of **55a** and **55b** were better than those of **56a** and **56b** and the activities of **55c** and **55d** were better than those of **56c** and **56d**.

**Scheme 15:**
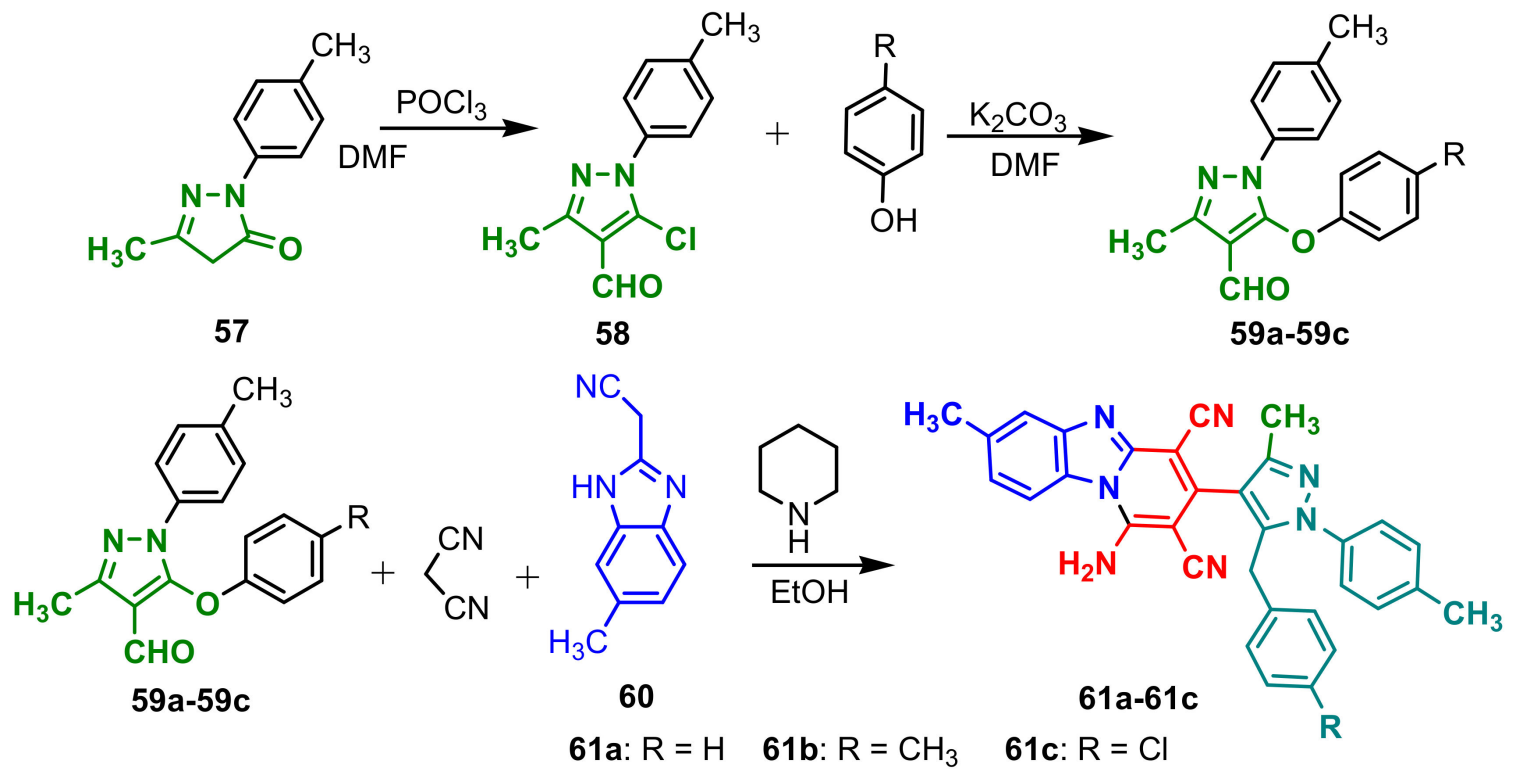
Synthesis of benzimidazole–pyrazole **61a**–**61c**.

Jardosh et al., synthesized new pyrido[1,2-a]benzimidazoles starting chloroformilation and alkilation of 4-methyl-2-*p*-tolylcyclopent-3-enone **57**. In the next step, a one-pot three-component reaction was used to afford final compounds **61a**–**61c** (Scheme 15). The in vitro antimicrobial activity of **61a**–**61c** against *S. typhi*, *S. pneumoniae*, *E. coli*, *C. tetani*, *V. cholera*, *B. subtilis*, *C. albicans* and *A. fumigatus* using broth microdilution technique was assessed. All compounds **61a**–**61c** displayed good antimicrobial activity compared to standard drugs, as can be seen in Table 5 [69]. 

**Scheme 16:**
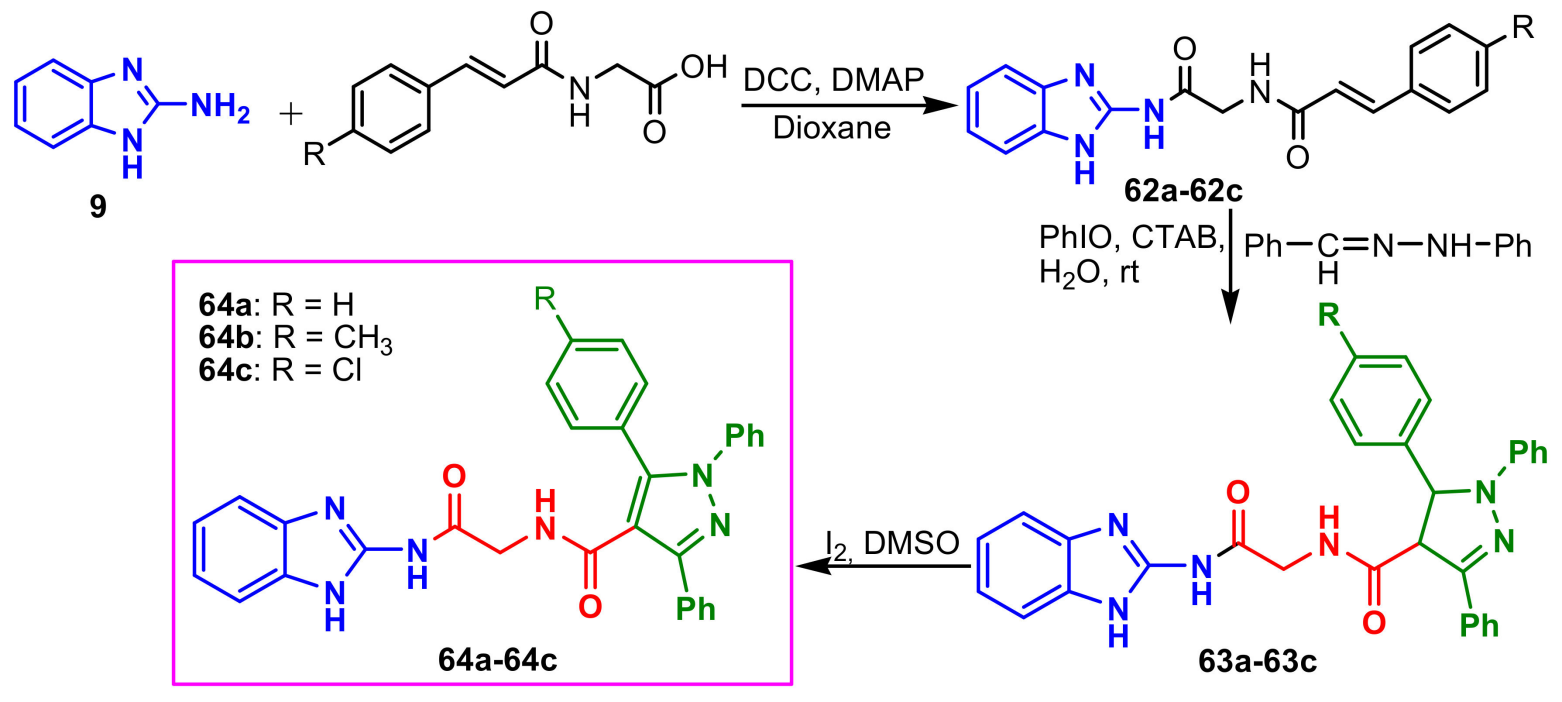
Synthesis of benzimidazole–pyrazole **64a**–**64c**.

Sowdari et al., synthesized a new class of diamidomethane-linked benzazolyl–pyrazoles **64a**–**64c** by a green approach, using the synthesis strategy indicated in Scheme 16 [70]. Compounds **64a** and **64c** were found to be potential antifungal agents against *Aspergillus niger* (MIC = 50 and 25 μg mL^−1^, respectively) and *Penicillium chrysogenum* (MIC = 12.5 and 12.5 μg mL^−1^, respectively) compared to the standard drug, Ketoconazole. 

**Scheme 17:**
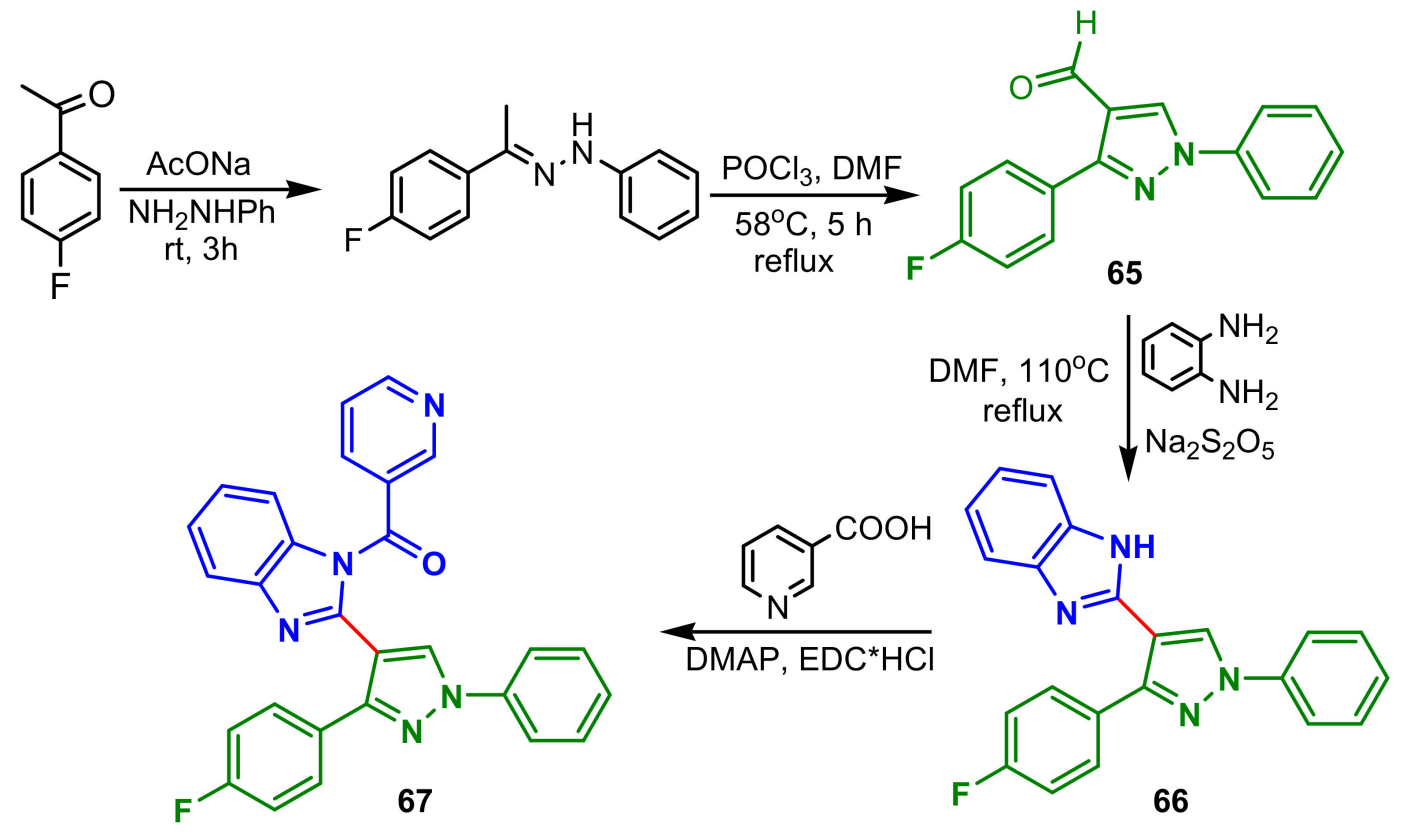
Synthesis of benzimidazole–pyrazole **67**.

β-ketoacyl-acyl carrier protein synthase III (FabH) is an attractive target for the development of new antibacterial agents, because it catalyzes the initial step of fatty acid biosynthesis, essential for bacterial survival. Thus, Wang et al., reported the synthesis of a new series of benzimidazole–pyrazol amides with low toxicity and potent FabH inhibitory. Synthesis of compound **67** from 1-(4-fluorophenyl)ethanone is accomplished in four steps: condensation with phenylhydrazine, followed by cyclization by reflux with POCl_3_ in DMF for 5 h, to obtain pyrazole **65**, which with 1,2-phenylenediamine and Na_2_S_2_O_5_ has provided benzimidazole–pyrazole intermediate **66**, which by acylation with nicotinic acid, DMAP and EDC hydrochloride led to the final product **67** (Scheme 17). Compound **67** showed the most potent inhibition activity against four bacteria strains (with MIC of 0.98, 0.49, 0.98, 0.98 μg mL^−1^, respectively, against *E. coli*, *P. aeruginosa*, *B. subtilis* and *S. aureus*) and FabH (with IC_50_ of 1.22 μM). Additionally, FabH mutant *Xanthomonas Campestris* experiment validated that compounds binding site outcomes FabH. 

**Figure 2:**
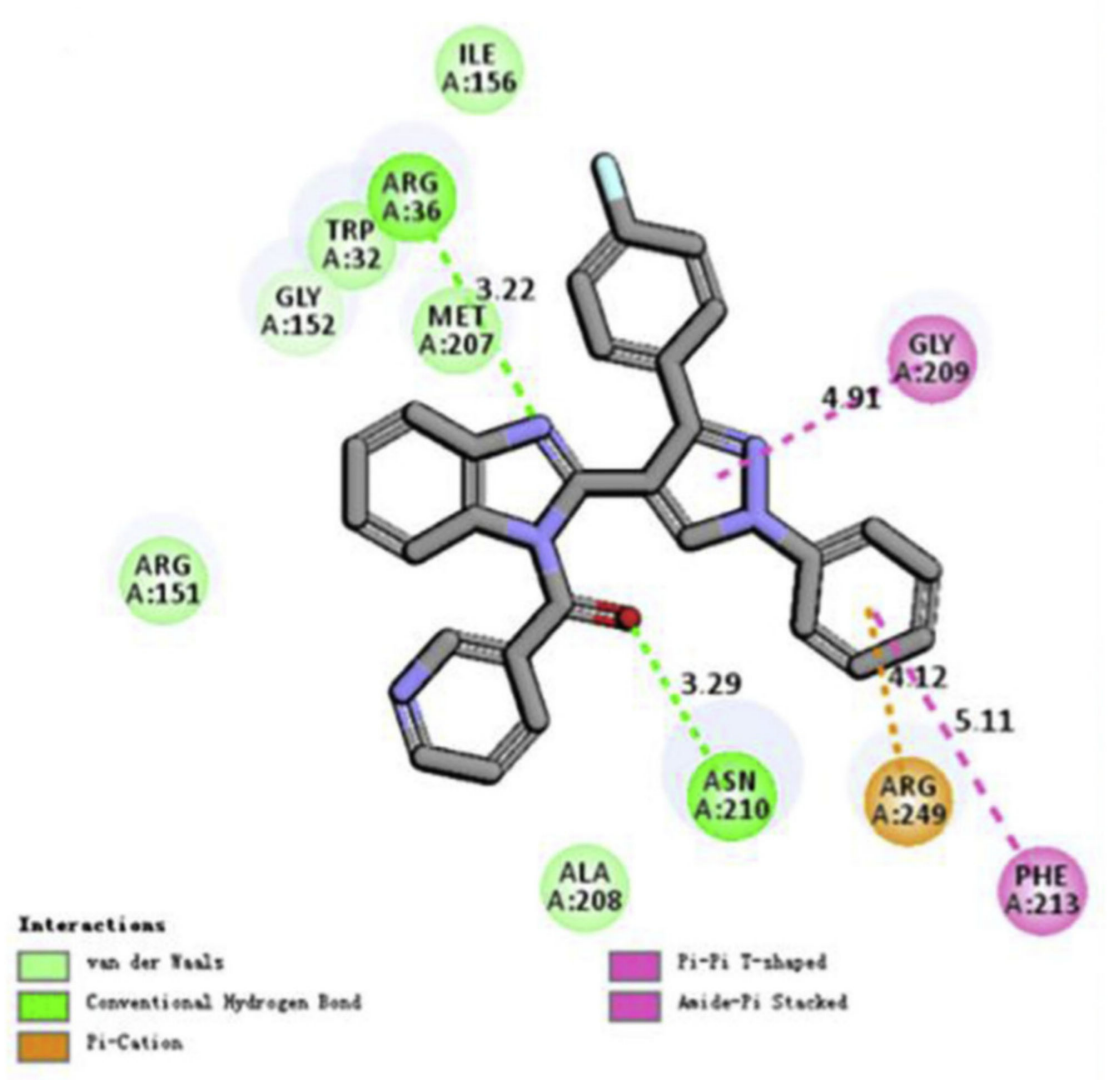
Docking model of representative compound **67**. 2D molecular docking modeling of compound **67** and surrounding residues of *E. coli* FabH (PDB code: 1HNJ) adapted from [71].

2D molecular docking modeling and surrounding residues of *E. coli* FabH was also performed for compound **67** (Figure 2) [71].

**Scheme 18:**
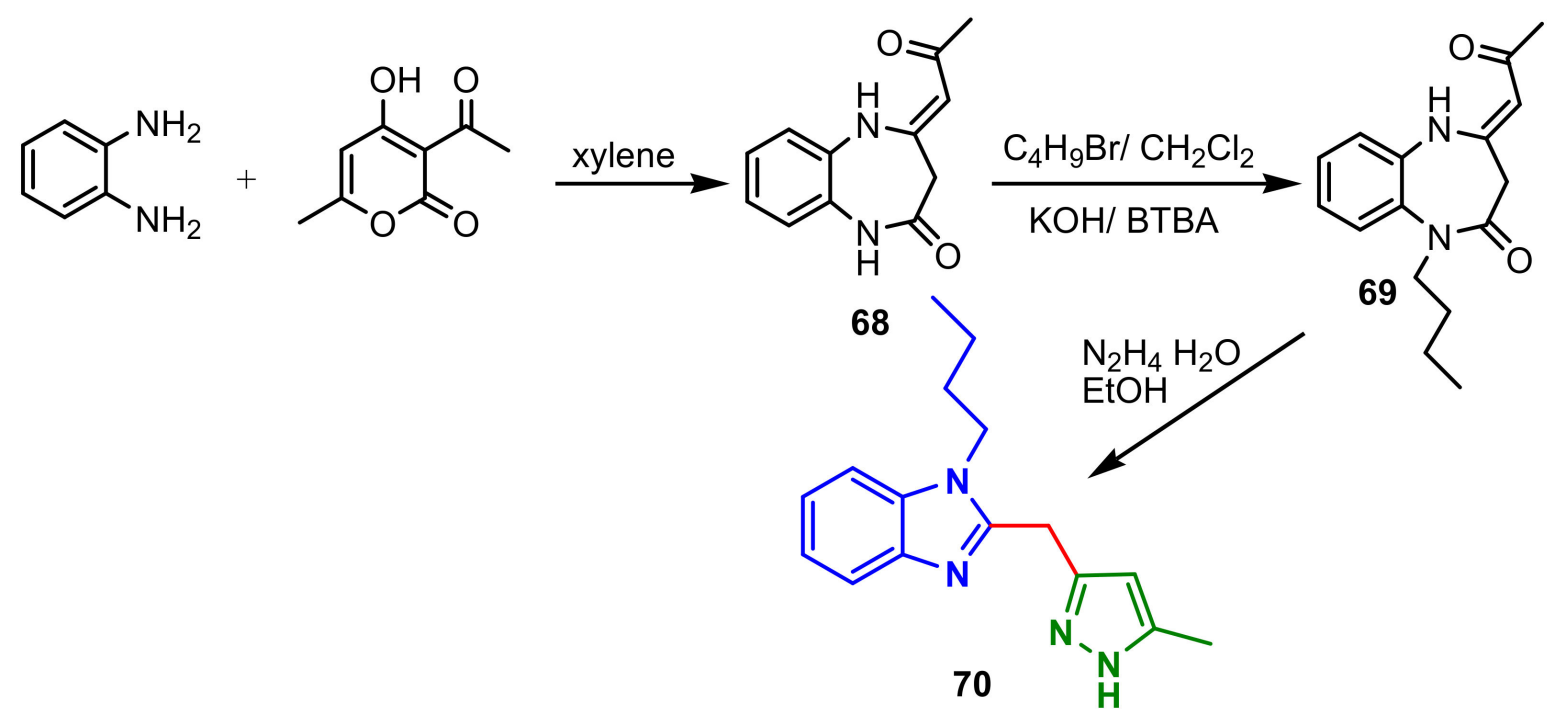
Synthesis of benzimidazole–pyrazole **70**.

A special method for the synthesis of a benzimidazolo–pyrazole compound was reported by Chkirate et al., [72]. Thus, condensation of 1,2-phenylenediamine with dehydroacetic acid afford **68** which reacted with 1-bromobutane to give the alkylated 1,5-benzodiazepine **69**. Compound **69** reacts with an excess of hydrazine monohydrate to afford the pyrazolyl–benzimidazole **70** (Scheme 18). The minimum inhibitory concentration (MIC) of **70** against *S. aureus*, *E. coli* and *P. aeruginosa* was evaluated at 12.5 μg mL^−1^, 50 μg mL^−1^ and 50 μg mL^−1^, respectively, compared to standard drug Chloramphenicol. Additionally, Co(II) and Zn(II) complexes of **70** possess remarkable antibacterial activity. 

**Scheme 19:**
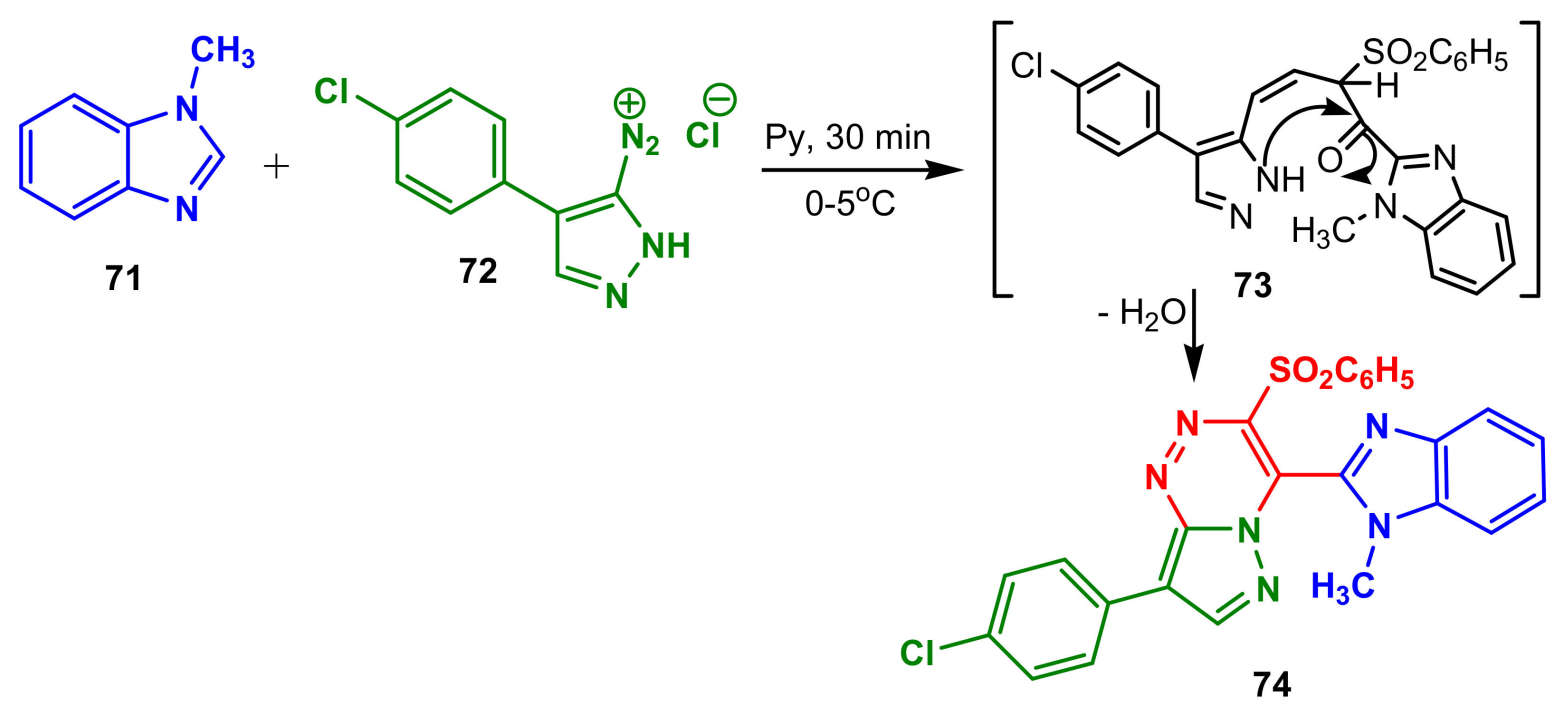
Synthesis of benzimidazole–pyrazole **74**.

Elaziz et al., synthesized benzimidazole–pyrazole **74** from 1-methylbenzimdazole **71** and diazonium salt **72**, through the intermediate **73 [73]**. Compound **74** possessed better antibacterial activity than standard Cephalothin against anaerobic *E. coli* (16.5 μg mL^−1^ versus 24.3 μg mL^−1^), *Salmonella typhimurium* (13.4 μg mL^−1^ versus 28.5 μg mL^−1^), and better antibacterial activity than standard Chloramphenicol against *Bacillus subtilis* (23.3 μg mL^−1^ versus 32.4 μg mL^−1^, Scheme 19, Table 6).

**Scheme 20:**
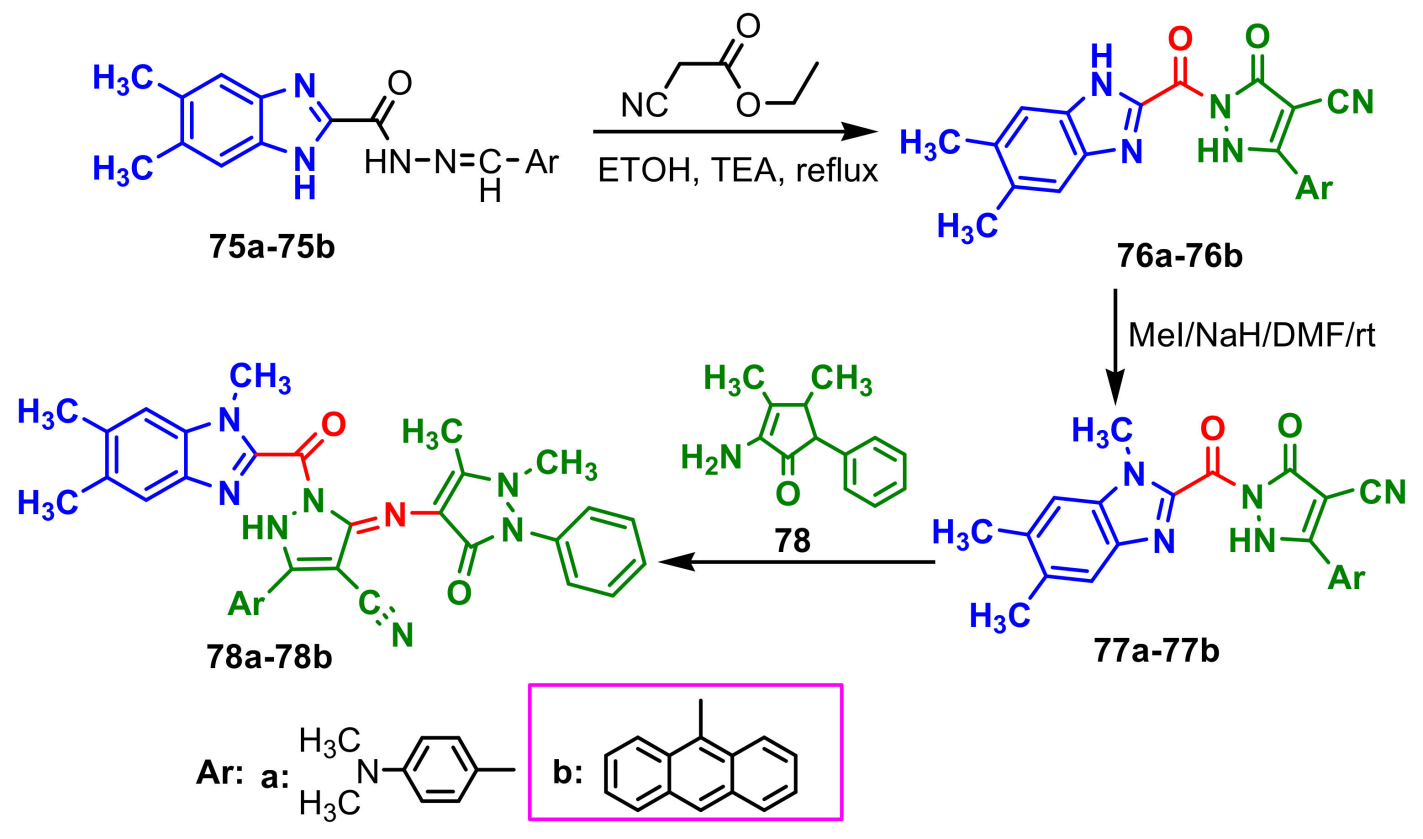
Synthesis of benzimidazole–pyrazoles **76a**–**76b**, **77a**–**77b**, **78a**–**78b**.

Bassyouni et al., synthesized three series of benzimidazole–pyrazoles **76**–**78b [74]**. Compounds **76a** and **76b** were synthesized by the reaction of **75a** and **75b** with ethyl cyanoacetate in ethanol in the presence of triethylamine, respectively (Scheme 20). Methylation of **76a** and **76b** was achieved by their reaction with methyl iodide or DMC that yielded compounds **77a** and **77b**. Compounds **77a** and **77b** reacted with 4-aminoantipyrine in ethanol, in the presence of catalytic amounts of acetic acid to give **78a** and **78b**. The antibacterial activity of the compounds **76a**, **76b**, **77b**, **78a** and **78b** was examined with Gram-positive bacteria *Bacillus subtilis*, *Bacillus cereus* and *Staphylococcus aureus*, Gram-negative bacteria *Escherichia coli*, *Pseudomonas aeruginosa* and *Salmonella* *typhimurium*. The antibacterial activity showed that compound **76a** was the most active against *S. typhimurium* and its activity exceeded the activity of the reference antibiotic amoxicillin. Compounds **77b** and **78b** exhibited high antimicrobial activity against *S. aureus* (Table 7).

**Scheme 21:**
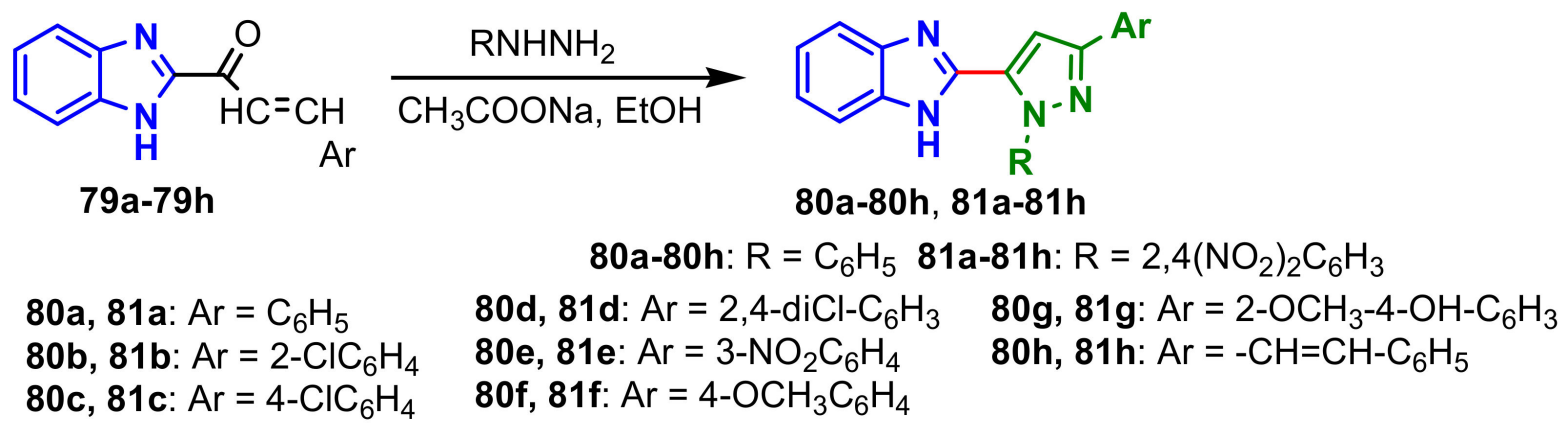
Synthesis of benzimidazole–pyrazoles **80a**–**80h**, **81a**–**81h**.

Benzimidazolo–pyrazole compounds **80a**–**80h** and **81a**–**81h** were synthesized from the reaction of chalcones **79a**–**79h** with phenylhydrazine and 2,4-dinitrophenylhydrazine, respectively (Scheme 21) [75]. All compounds were screened for their antimicrobial activities against *E. coli*, *P. aeruginosa*, *S. aureus*, *B. subtilis*, *C. albicans* and *A. niger* (Table 8). The best antimicrobial activities of the compounds are marked in green in Table 8. It is observed that compounds **80b** and **80h** showed a good antibacterial activity against all the strains tested, and compounds with 2,4-dinitrophenylhydrazine had better antifungal activity than the antibacterial one, e.g., compounds **81b** and **81f**. Only compound **80a** showed significant antitubercular activity at the concentration of 100μg mL^−1^ compared with the standard drug, Rifampicin.

**Scheme 22:**
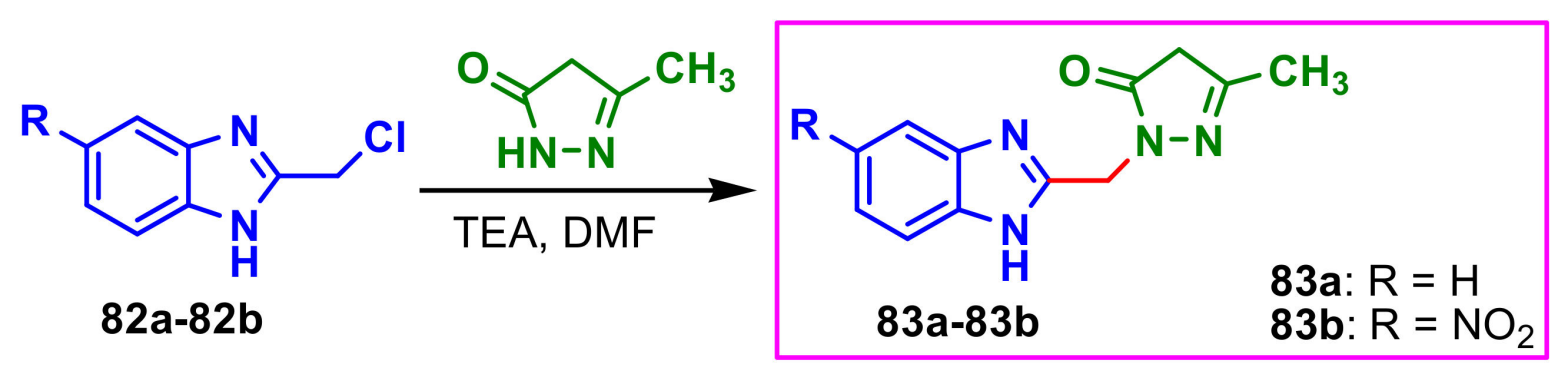
Synthesis of benzimidazole–pyrazoles **83a**–**83b**.

El-Gohary et al., synthesized benzimidazole–pyrazole molecules **83a**–**83b** with antimicrobial properties, using the reaction between benzimidazoles **82a**–**82b** and 3-methyl-1*H*-pyrazol-5(4*H*)-one in dimethylformamide (DMF), in presence of triethyl-amine (TEA) as catalyst (Scheme 22) [76]. The compounds **83a**–**83b** showed very good antimicrobial activity against two bacteria *B. cereus* and *S. aureus*, against two fungi, *C. albicans* and *A.* *fumigatus*, compared to the standards used, ampicillin and fluconazole (Table 9).

**Scheme 23:**
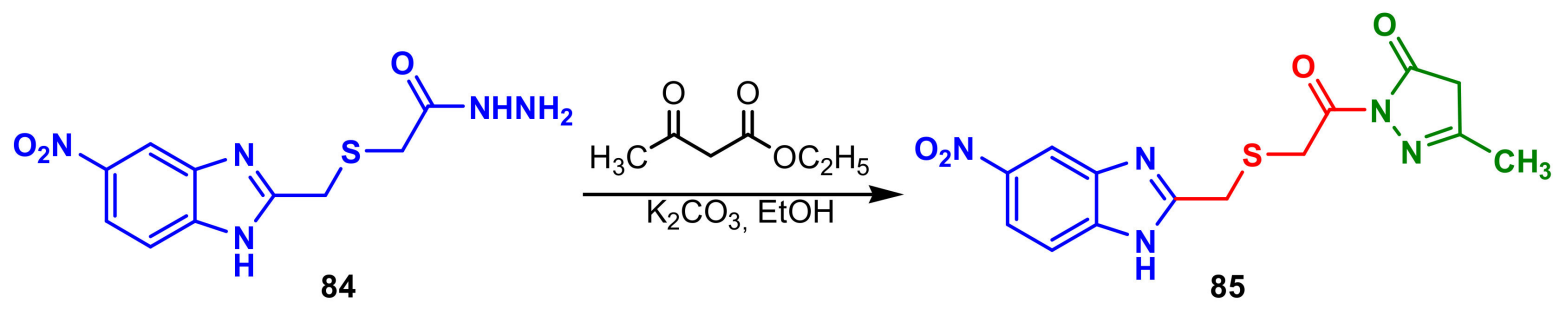
Synthesis of benzimidazole–pyrazoles **85**.

The benzimidazole–pyrazole **85** synthesized by cyclization of benzimidazole **84** in the reaction with of ethyl 3-oxobutanoate (Scheme 23), possessed good antifungal activity, against *C. albicans* (MIC = 2500 μg mL^−1^) compared with standard Fluconazole (MIC = 2500 μg mL^−1^) [77]. 

### 2.2. Benzimidazoles Substituted in the Position “1” with Pyrazole Moiety

**Scheme 24:**
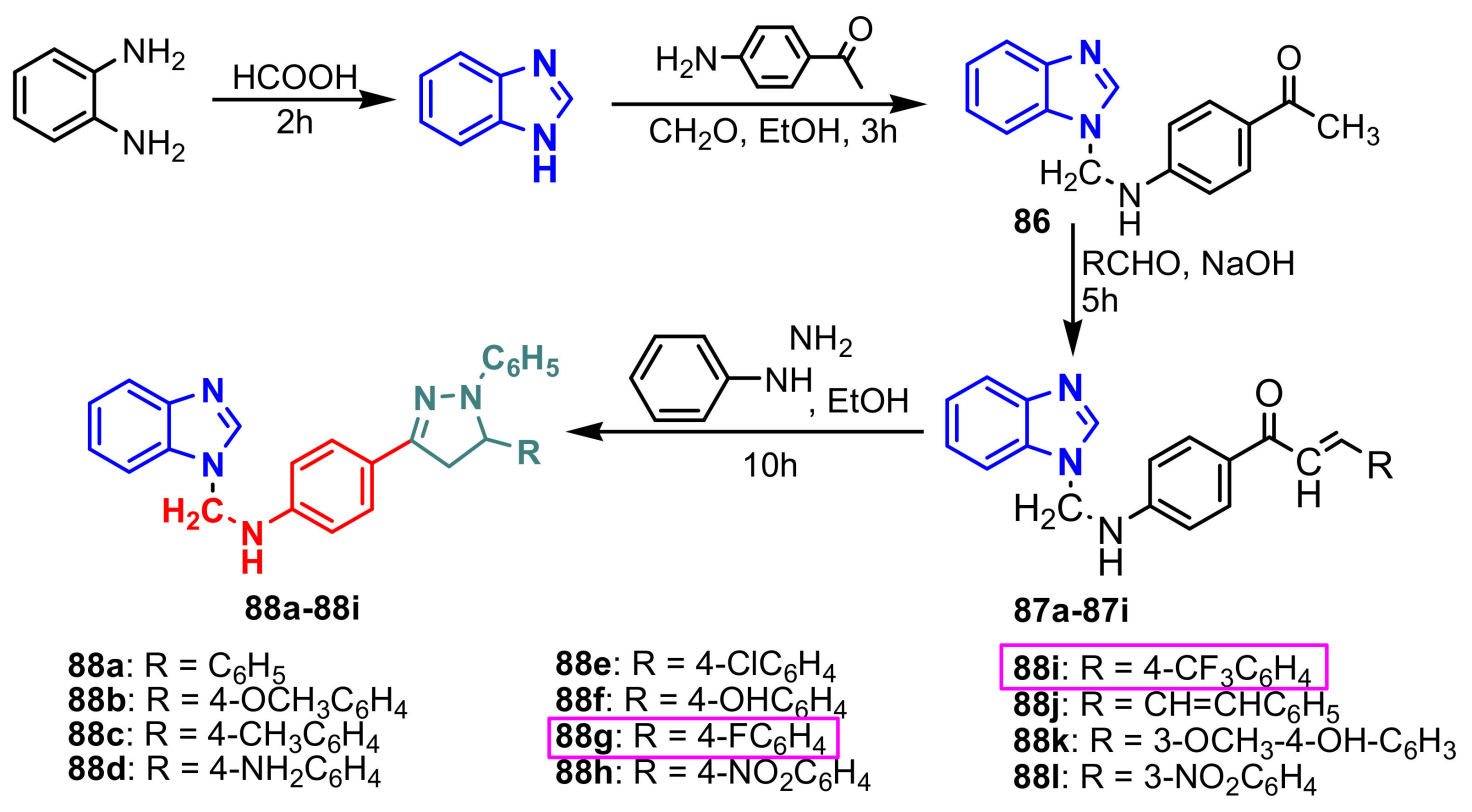
Synthesis of benzimidazole–pyrazole **88a**–**88i**.

Krishnanjaneyulu et al., synthesized a series of 1-substituted benzimidazoles with pyrazole moiety through a linker using a four-step strategy: benzimidazole synthesis, N-alkylation, condensation with aldehydes with the formation of chalcones **87a**–**87i** and cyclization with the formation of the pyrazole nucleus, in compounds **88a**–**88i** (Scheme 24) [78]. All compounds were evaluated for their antibacterial activity against four Gram-positive bacteria: *Staphylococcus aureus* ATCC 9144, *Staphylococcus epidermidis* ATCC 155, *Micrococcus luteus* ATCC 4698 and *Bacillus cereus* ATCC 11778, and three Gram-negative bacteria: *Escherichia coli* ATCC 25922, *Pseudomonas aeruginosa* ATCC 2853 and *Klebsiella pneumoniae* ATCC 11298. The antifungal activity of the compounds **88a**–**88i** was evaluated against two fungi, *Aspergillus niger* ATCC 9029 and *Aspergillus fumigatus* ATCC 46645 (Table 10). It was found that compounds containing an electron-withdrawing group at the phenyl group attached to C-5 of pyrazole displayed superior antimicrobial activities than compounds possessing an electron-releasing group. The unsubstituted derivatives displayed moderate antimicrobial activity. The best antimicrobial activity was shown by compounds **88g** and **88i**, which in Table 10 is marked in green.

**Scheme 25:**
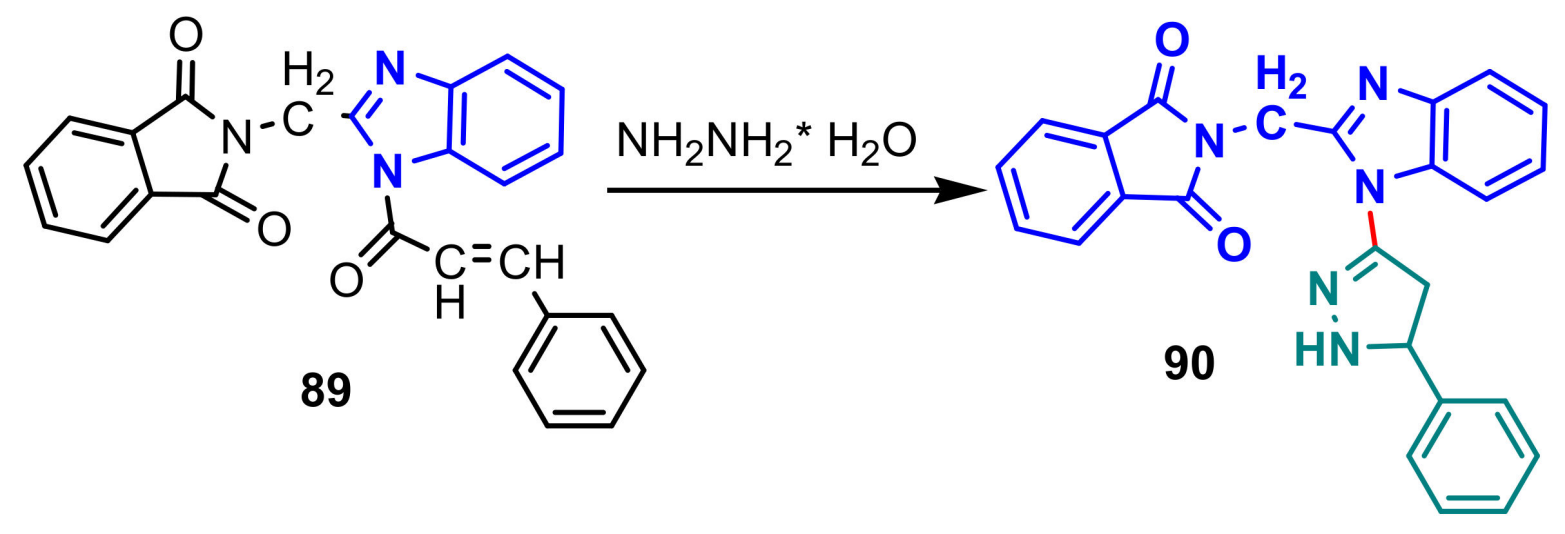
Synthesis of benzimidazole–pyrazole **90**.

Dawoud et al., reported the synthesis of a benzimidazole–pyrazole compound **90** from chalcone intermediate **89** (Scheme 25). It was found by the agar diffusion method that compound **90** possessed good antimicrobial activity against *Escherichia coli*, *Salmonella. SP*., *Staphylococcus aurous* and *Candida albicans* [79].

**Scheme 26:**
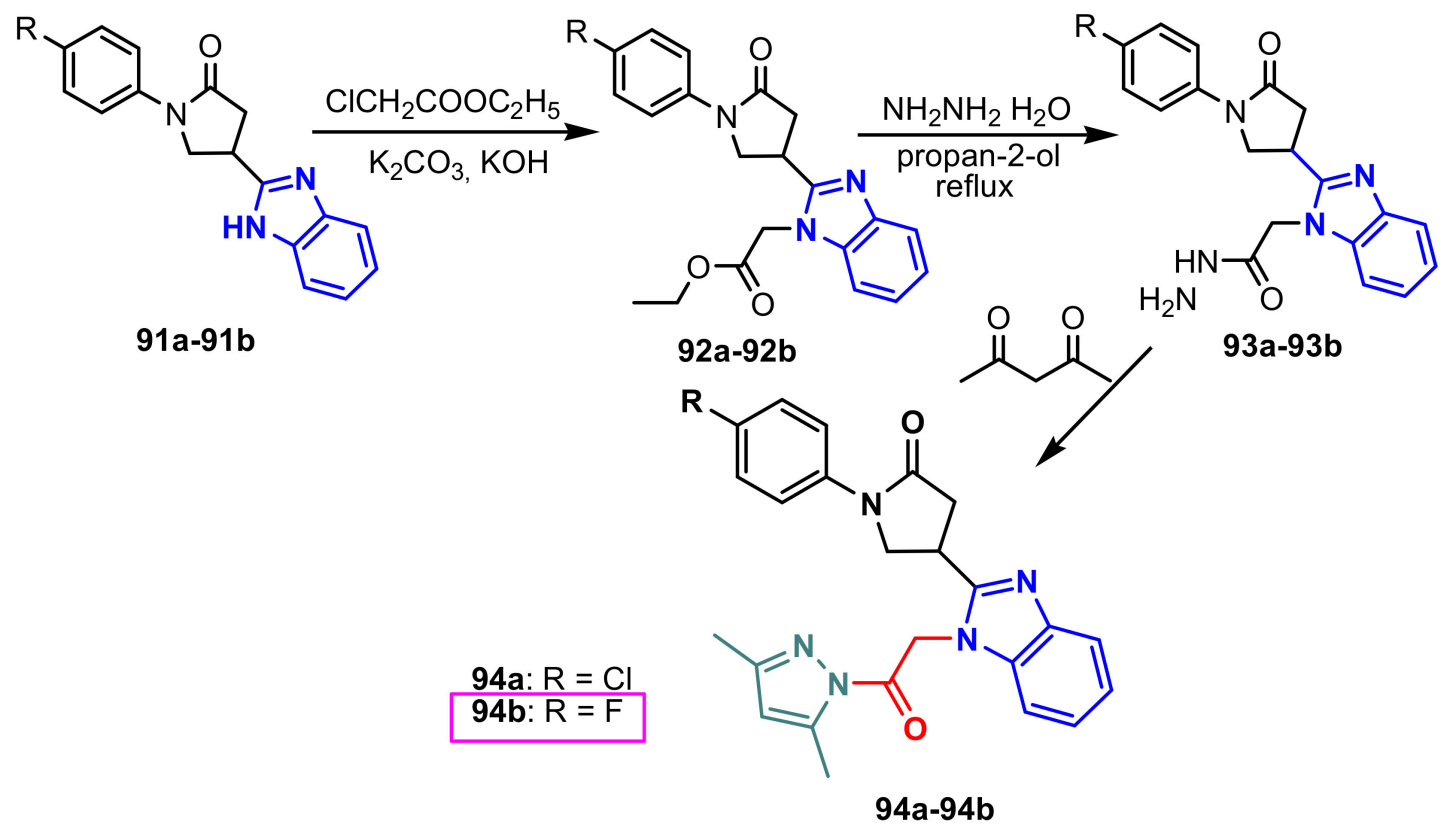
Synthesis of benzimidazole–pyrazoles **94a**–**94b**.

Tumosienė et al., reported the synthesis of the benzimidazole–pyrazole compounds **94a**–**94b**, in three steps, from 2-substituted benzimidazoles **91a**–**91b** (Scheme 26). It was found that compound **94b** shows good antimicrobial activity against *Staphylococcus aureus* ATCC 9144 and *Escherichia coli* ATCC 8739 (250 μg mL^−1^) [80].

### 2.3. Benzimidazoles Substituted in the Position “4” (“7”) with Pyrazole Moiety

**Scheme 27:**
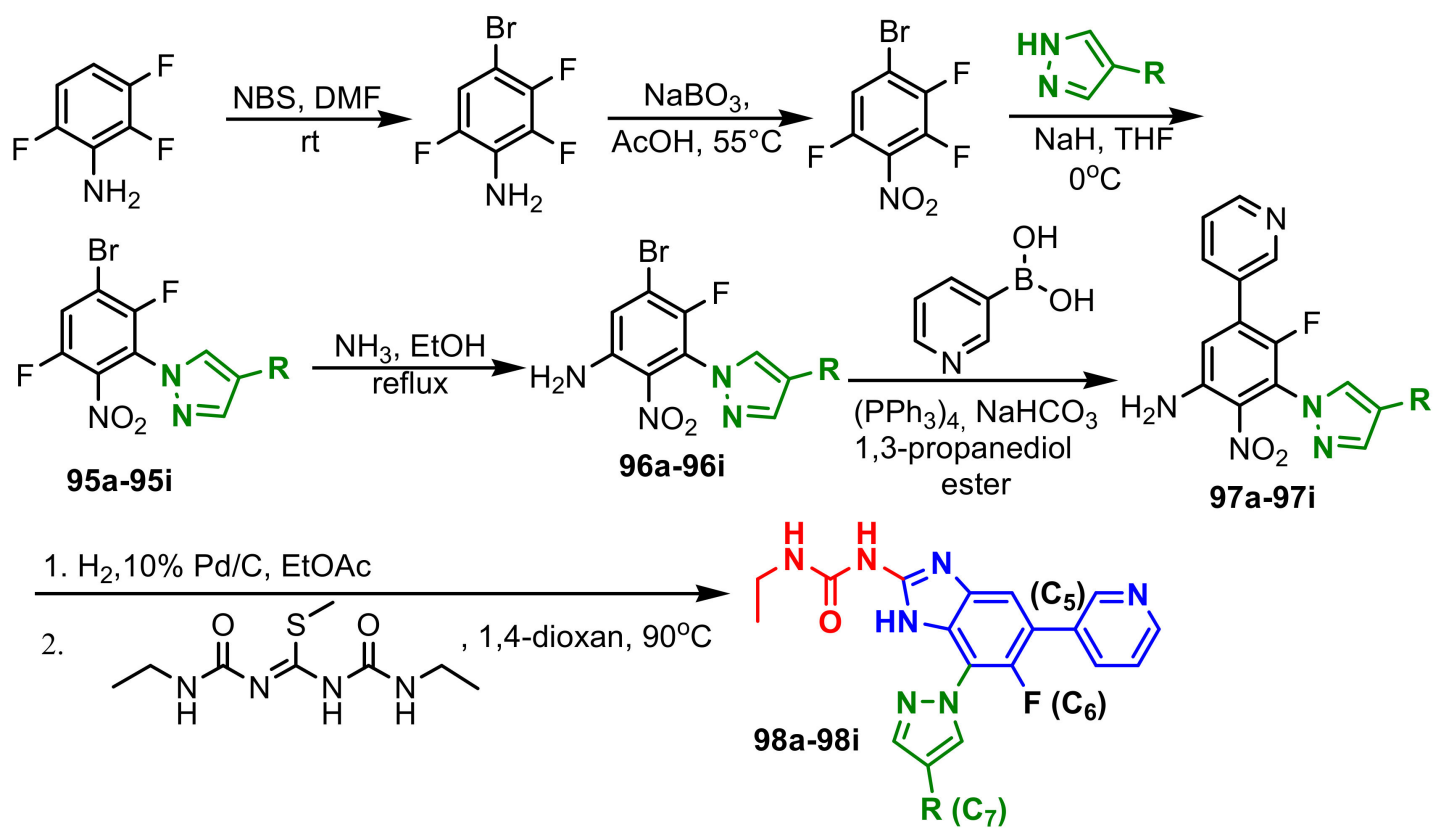
Synthesis of benzimidazole–pyrazoles **98a**–**98i**.

Grilot et al., reported the synthesis of second-generation antibacterial benzimidazole–pyrazoles from 2,3,6-trifluorobenzenamine in seven steps, as can be seen in Scheme 27 [81]. Compound **98a**, with a 3-pyridine moiety at C5, which maintains a hydrogen bond with Arg136, showed a reasonable MIC against *S. aureus* (0.25 μg mL^−1^). It can be observed that introduction of a fluorine atom at C6 on pyrazole **98b**, has no improved antibacterial potency (0.25 μg mL^−1^ against *S. aureus*), nor affinity for Gyrase B and Topoisomerase IV, as previously reported [80], but oral exposure was improved 2-fold, which led to the exclusive focus on C6-fluorobenzimidazole pyrazoles. The authors also studied the variation of MIC and the polarity of molecules with the introduction of the substituent in the “5” position, as seen in Table 11. Compound **98f** showed a MIC against *S. aureus* of 0.125 µg mL^−1^ and could be improved slightly by the addition of a methyl group on the C5 substituent, as in **98g** and its resolution yielded compounds **98h** and **98i**. The (*S*)-isomer **98i** was 4-fold more potent than the (*R*)-isomer **98h** against *S. aureus* and showed acceptable oral exposure, but compound **98h** exhibited a high serum shift (16-fold).

**Scheme 28:**
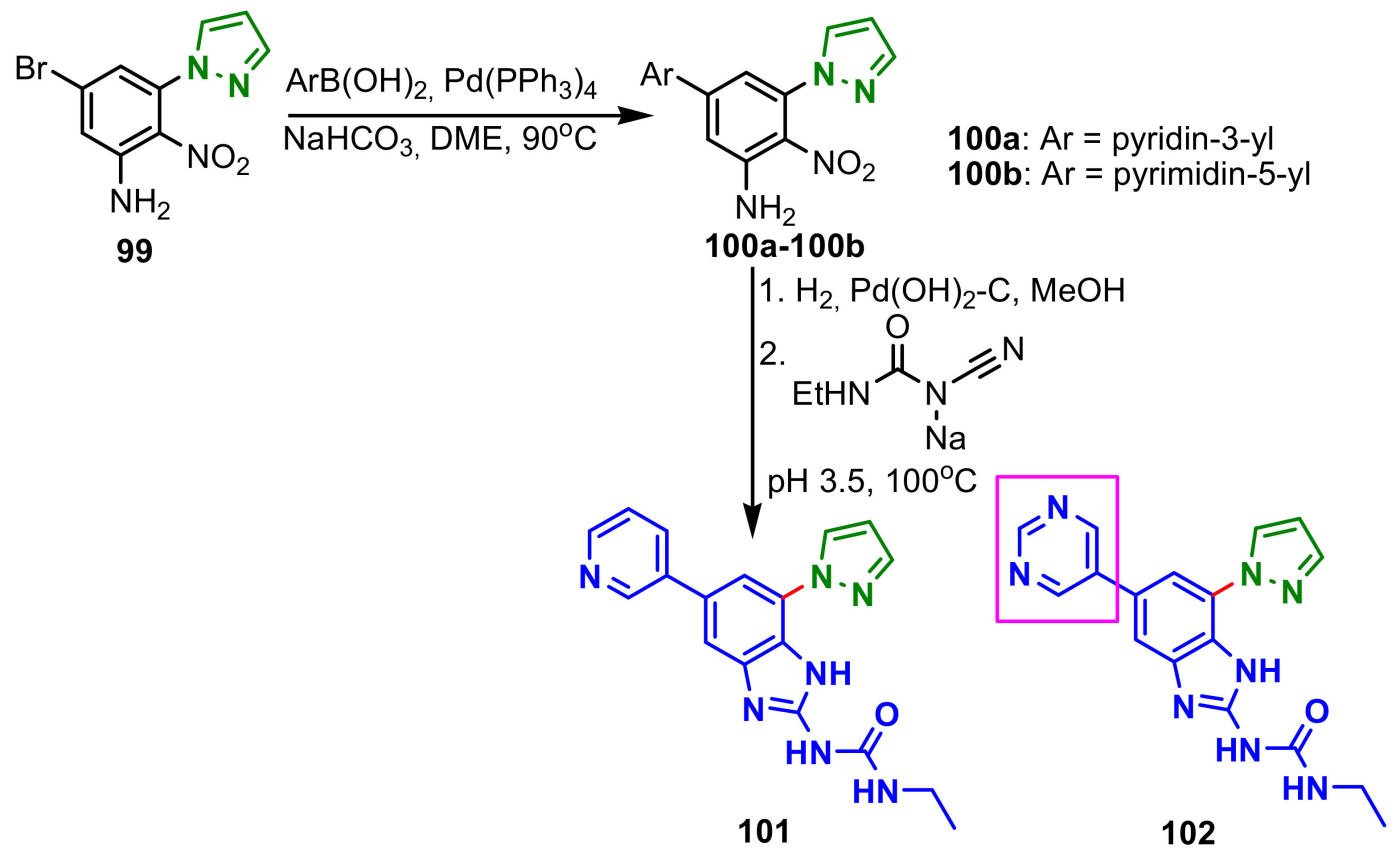
Synthesis of benzimidazole–pyrazoles **101** and **102**.

Charifson et al., reported the synthesis of benzimidazole–pyrazoles **101**–**102** from 5-bromo-2-nitro-3-(1*H*-pyrazol-1-yl)benzenamine **99**, using a Suzuki coupling reaction (Scheme 28) [82]. The compounds were found to be inhibitors of DNA Gyrase and Topoisomerase IV, with potent antibacterial activity. The results of the evaluation for enzymatic inhibition and antibacterial potency of the compounds are shown in Table 12. The superior enzymatic and antibacterial inhibitory activity of compound **102** vs. **101** is observed, due to the presence of the pyrimidine nucleus in the molecule.

## 3. Biological Activities of Antimicrobial Benzimidazole–Pyrazole Compounds

### 3.1. Analgesic and Anti-Inflammatory Activity of Antimicrobial Benzimidazole–Pyrazole Compounds

Benzimidazole and pyrazole/pyrazoline are important nitrogen-containing heterocyclics in anti-inflammatory research [83,84]. In recent decades, there have been various studies on the anti-inflammatory activity of benzimidazole compounds.

Chikkula et al., reported the analgesic activity [65] of the compounds **39a**–**39i** (Scheme 11). The analgesic activity of novel benzimidazole derivatives **39a**–**39i** varied with reaction time. The compounds displayed moderate analgesic activity at 30 min of reaction time. In the second hour, the analgesic activity reached to peak level. Additionally, the presence of phenyl ring at the N-1 atom of pyrazole ring significantly increased analgesic activity. The anti-inflammatory activity of the synthesized compounds varies similarly to their analgesic activity. Compounds **39c**, **39d** and **39e** had the best analgesic activity.

**Scheme 29:**
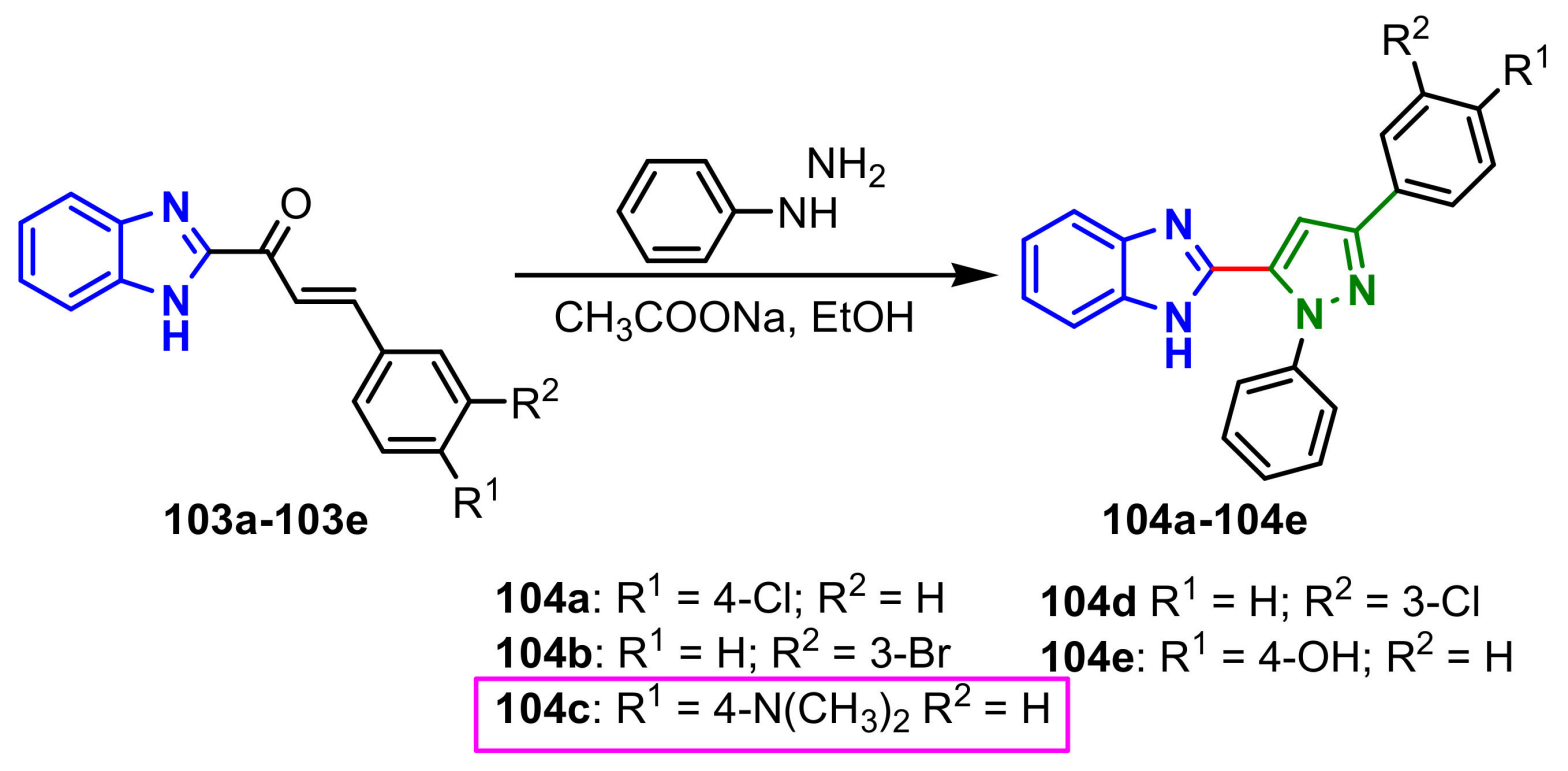
Synthesis of benzimidazole–pyrazoles **104a**–**104e**.

Arora et al., reported the synthesis of the compounds **104a**–**104e** from chalcones **103a**–**103e** (Scheme 29) [85]. The in vivo studies for analgesic activity were evaluated in albino mice by Eddy’s hot plate method. The compound **104a** showed mild activity at a dose of 100 and 200 mg kg^−1^ in the range of 7.38 ± 0.12–7.36 ± 0.15 at 90 min when compared with standard drug Diclofenac sodium at a dose of 5 mg kg^−1^ in the range of 9.45 ± 0.28 at 90 min. Additionally, the compound **104a** showed slightly less moderate activity in the range of 53.03 and 59.09% at a dose of 100 and 200 mg kg^−1^ at a time interval of 3h of carrageenan challenge when compared to compound **104c** (63.63%) as well as when compared with standard Diclofenac sodium at the same time period exhibited 69% of activity, respectively, at a dose of 100 mg kg^−1^.

### 3.2. Benzimidazole–Pyrazole Compounds with Antitumor Activities 

Kalirajan et al., [75] reported anticancer activity of compounds **80a**–**80h** and **81a**–**81h** against MCF7 human breast cell line by in vitro Sulforhodamine B assay (SRB assay) method. The compounds **80b**, **81a** and **81b** have significant activity when compared with standard drug Doxorubicin (Adriamycin, ADR), with GI_50_ values of 16.3 μg mL^−1^, 16.0 μg mL^−1^, and 17.1 μg mL^−1^, respectively (Doxorubicin with GI_50_ value <10 μg mL^−1^). El-Gohary and Shaaban [76] reported the antitumor activity of compounds **83a** and **83b** and the results are shown in Table 13. Compound **83a**, exhibiting the highest in vitro antitumor activity, was evaluated for in vivo antitumor activity against EAC in mice. Additionally, compound **83a** was assessed for in vitro cytotoxicity toward human normal lung fibroblast (W138) cell line employing MTT assay [86,87,88] and utilizing 5-fluorouracil as a standard cytotoxic drug. IC_50_ value for **83a** was determined 0.246 mM, therefore less cytotoxic than 5-fluorouracil (IC_50_ = 0.051 mM). Additionally, results of the DNA-binding assay confirmed that antimicrobial and antitumor compound **83a** exerts its biological activities through interaction with DNA. Bezimidazole–pyrazole **85** displayed eminent activity toward HCT-116 cell line [77] with IC_50_ value of 5.41 μM close to that of 5-fluorouracil of 4.00 μM and lower than that measured for the previously prepared benzimidazoles [76,89].

**Scheme 30:**
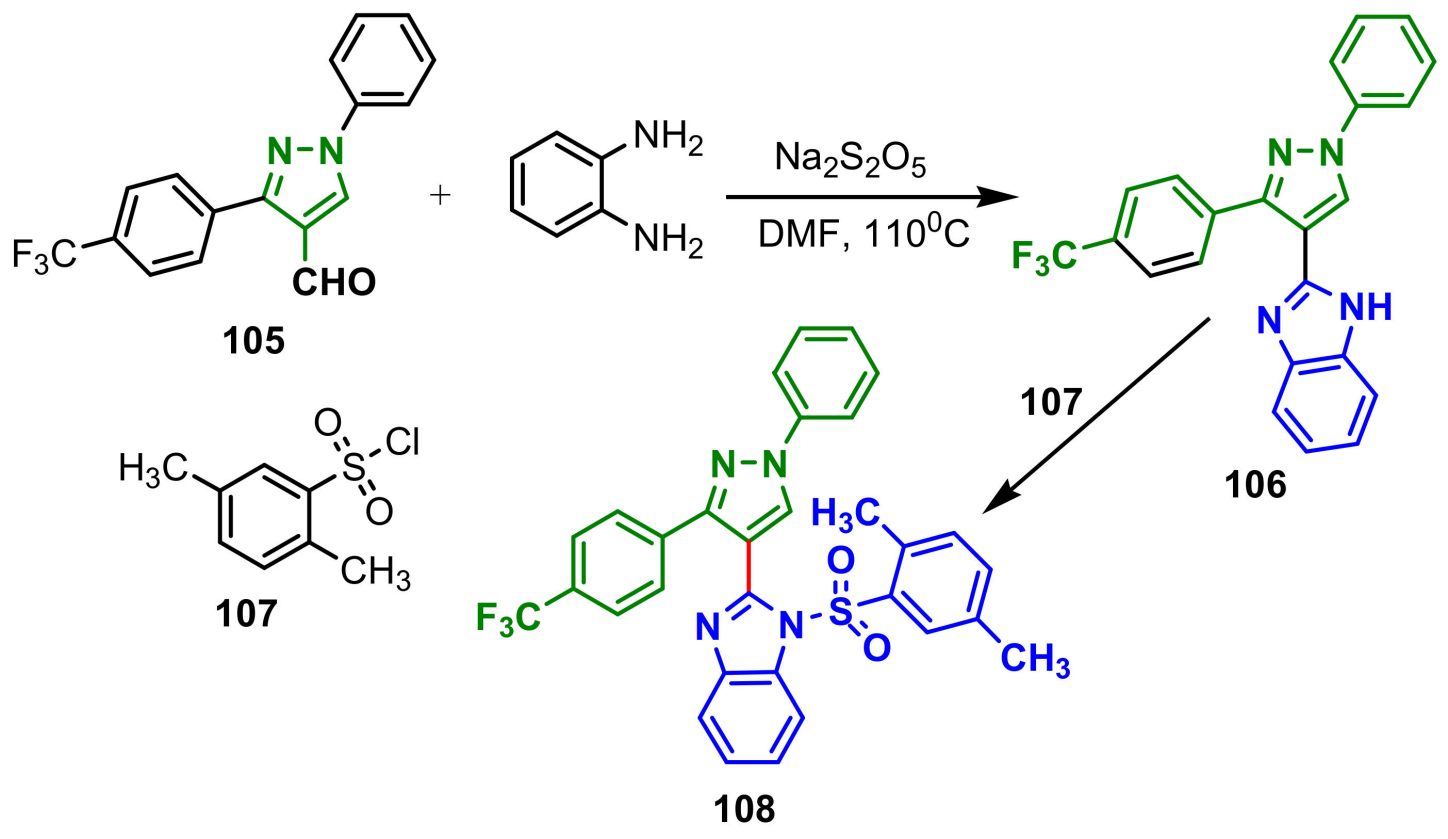
Synthesis of benzimidazole–pyrazole **108**.

Wang et al., synthesized a series of benzimidazole grafted benzsulfamide-containing pyrazole ring derivatives using a strategy in two steps: 1. synthesis of benzimidazole–pyrazole hybrid by reaction of pirazolecarbaldehyde **105** with 1,2,-phenylenediamine and 2. reaction with sulfonyl chloride **107** with the hybrid **106** (Scheme 30) [90]. Compound **108** showed the most excellent inhibition against tubulin assembly (IC_50_ = 1.52 μM) and in vitro growth inhibitory activity against a panel of four human cancer cell lines, IC_50_ = 0.15, 0.21, 0.33 and 0.17 μM, respectively, for A549, Hela, HepG2 and MCF-7. Additionally, compound **108** validly induces A549 cell apoptosis, causes cell cycle arrest in the G2/M phase and disrupts the cellular microtubule network. Due to these promising results, along with molecular docking data, compound **108** is a potential anticancer agent.

**Figure 3:**
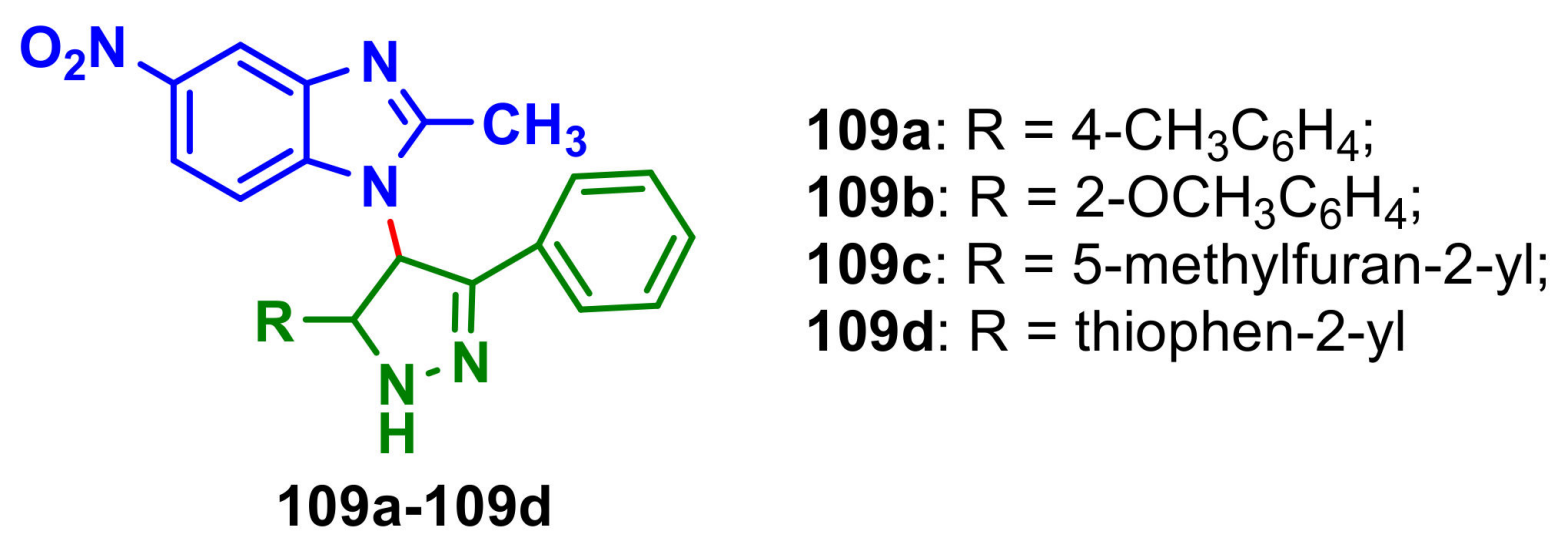
Benzimidazole–pyrazoles **109a**–**109d**.

Shake et al., reported synthesis of 1-substituted benzimidazoles with pyrazole moiety **109a**–**109d** (Figure 3), by cyclization of the corresponding chalcones with hydrazine hydrate in ethanol, and their antitumor and antiviral activities [91]. The in vitro cytotoxic screening of the compounds **109a**–**109d** against four different cell lines is showed in Table 14. It can be seen that compound **109d** has the best antitumor activity on all determined tumor lines.

### 3.3. Benzimidazole–Pyrazole Compounds as Antioxidants

Saundane et al., evaluated the scavenging effects of the compounds **31a**–**31c** and **32a**–**32c** [63] on the DPPH radical by Hatano’s method [92]. The RSA (Radical Scavenging Activity) results suggested that the compound **31a** exhibited good antioxidant activity of 71.95 and 72.43 % at the concentration of 100 μg mL^−1^. Additionally, the reductive ability of synthesized compounds was assessed by the extent of conversion of Fe^3+/^ferricyanide complex to the Fe^2+/^ferrous form. The reductive ability results suggested that the compound **31a** exhibited good reducing power ability at the concentration of 100 μg mL^−1^. 

Bassyouni et al., reported that compounds **77a**, **77b** and **78b** displayed mild antioxidant activity, of 227.9 μmol L^−1^, 412.7 μmol L^−1^ and 361.8 μmol L^−1^, respectively, using the 2,2-diphenyl-1-picrylhydrazyl radical scavenging assay [74].

**Figure 4:**
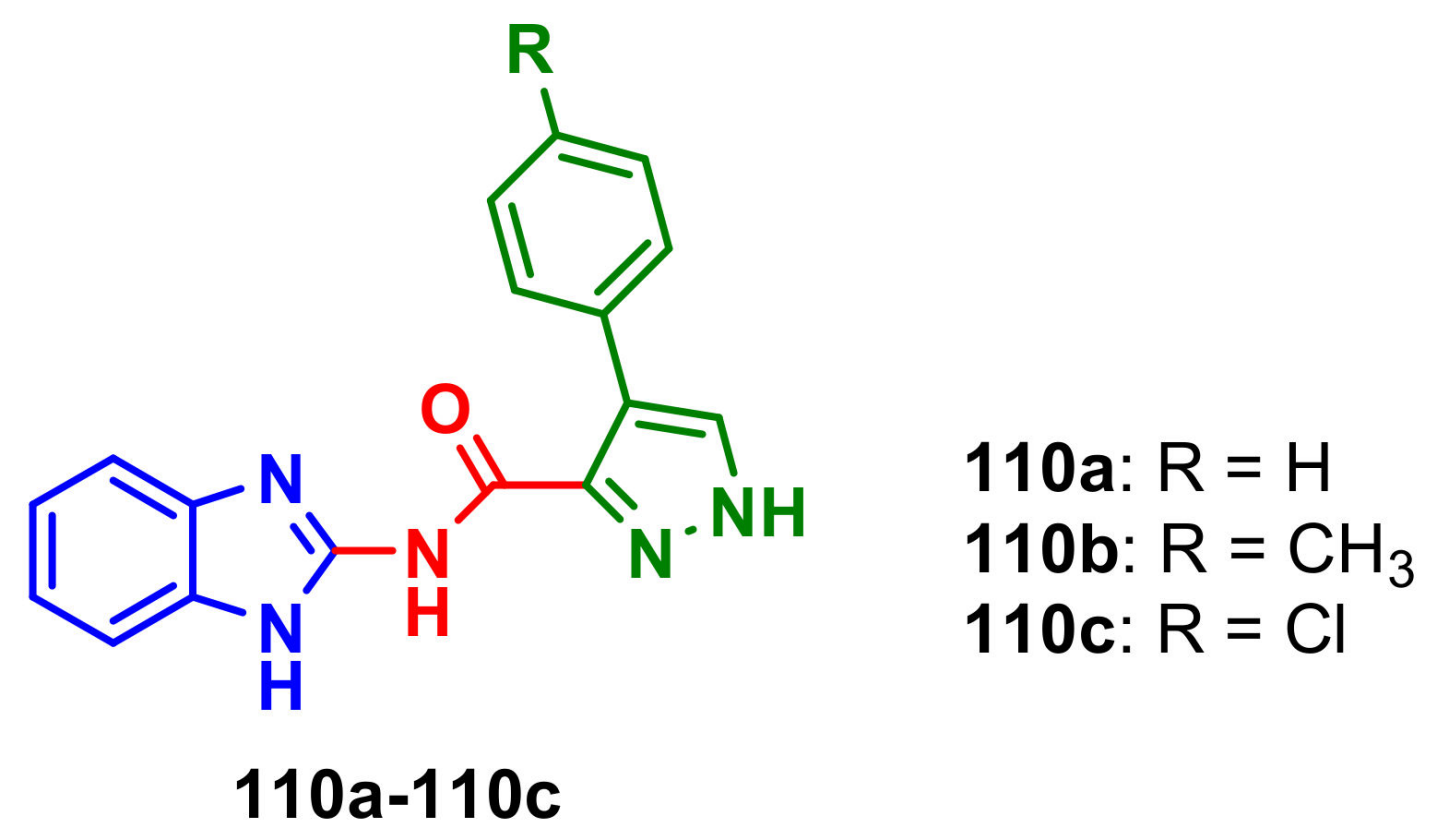
Benzimidazole–pyrazoles **109a**–**109d**.

Durgamma et al., reported the synthesis of amido-linked benzimidazolyl–pyrazoles **110a**–**110c** (Figure 4) from the corresponding chalcones [93]. All compounds possess good antioxidant activity in the DPPH method, with IC_50_ values of 0.121 μmol mL^−1^, 0.127 μmol mL^−1^ and 0.135 μmol mL^−1^, respectively, compared to ascorbic acid with IC_50_ = 0.256 μmol mL^−1^.

### 3.4. Benzimidazole–Pyrazole Compounds as Antiulcer Agents

**Scheme 31:**
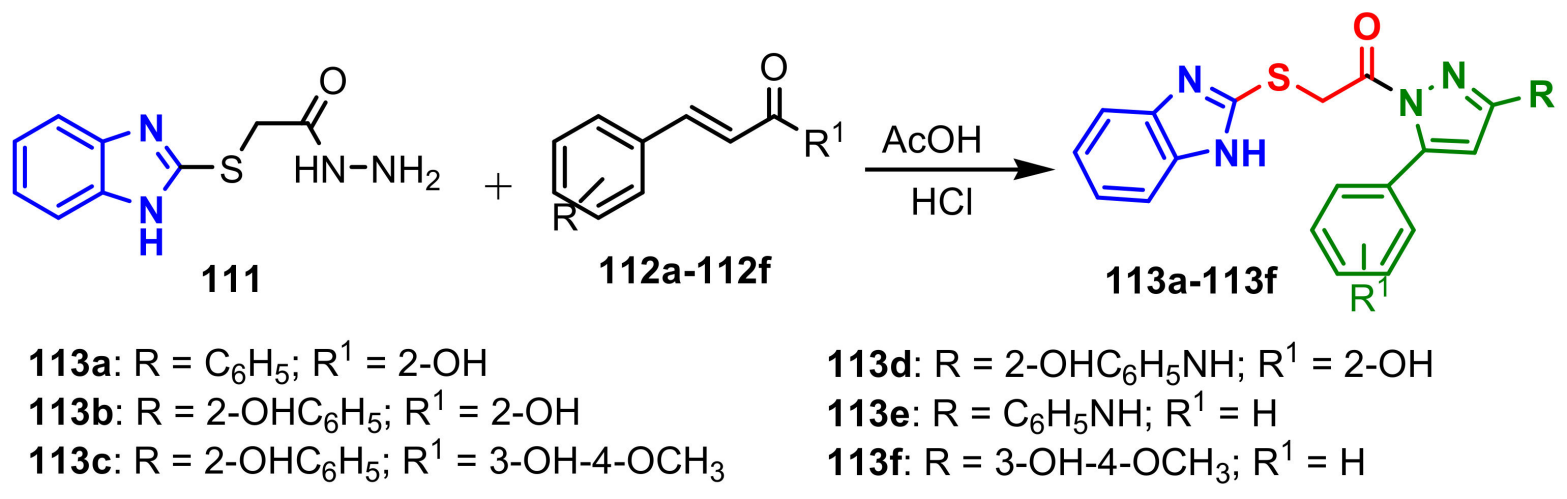
Synthesis of benzimidazole–pyrazoles **113a**–**113f**.

Noor et al., synthesized the benzimidazole–pyrazole hybrids **113a**–**113f** by reaction of benzimidazole–hydrazide **111** with chalcones **112a**–**112f** in acetic acid (Scheme 31) [94]. The antiulcer activity of all the benzimidazole–pyrazole hybrids was tested in vivo by an ethanol-induced gastric ulcer model in rats, with Omeprazole (30 mg/kg) as a standard. All compounds **113a**–**113f** exhibited an antiulcer effect. 

## 4. Conclusions

This review summarizes the syntheses of benimidazole–pyrazole compounds with antimicrobial properties, as well as their biological activities mentioned in the literature. From the data presented, it can be concluded that hybrids with pyrazole moiety in position “4” (“7”) possess the strongest antimicrobial properties. The presence of certain groups grafted on the benzimidazole and pyrazole nuclei, such as -COOCH_3_, -NHCO, -CHO, -CF_3_, -NO_2_, -CN, -F, -Cl, -OH, OCH_3_, -N(CH_3_)_2_ as well as other heterocycles in the molecule (pyridine, pyrimidine, thiazole, indole), increases the antimicrobial activity of the compounds [95]. Additionally, the binding linker between benzimidazole and pyrazole is important for their antimicrobial activity. Additionally, the antimicrobial activity is improved if the molecule contains linker groups such as carbonyl (CO), amide (NHCO), or other heteroatoms. We hope that this review will be a starting point for the synthesis of other benzimidazole–pyrazole hybrids with antimicrobial properties, which have much better bacterial and antifungal properties than those of antibiotics marketed or used in hospitals today.

## Figures and Tables

**Table 1:** Antibacterial and antifungal activities of the compounds **28a**–**28h** indicated by MIC (μg mL^−1^).

Compound	*E. coli*	*S. aureus*	*C. albicans*	*A. niger*
**28b**	312	312	312	312
**28c**	187.5	312	312	312
**28d**	250	62.5	250	187.5
**28e**	187.5	62.5	312	250
**28f**	312	187.5	62.5	312
**28g**	62.5	62.5	312	312
**28h**	125	62.5	62.5	125
Streptomycin	125	125	-	-
Fluconazole	-	-	125	125

**Table 2:** Antibacterial and antifungal activities of the compounds **28a**–**28h**.

Compound	Antibacterial Activity (MIC μg mL^−1^)				Antifungal Activity (MIC μg mL^−1^)			
	Ec	Sa	Kp	Pa	Ao	An	Af	At
**31a**	8	8	16	16	16	8	16	8
**31b**	32	64	128	128	64	128	256	128
**31c**	128	512	512	256	512	512	256	128
**32a**	8	16	16	32	32	16	8	32
**32b**	32	32	16	128	128	128	256	128
**32c**	32	64	512	256	128	256	256	128
Gentamycin	2	2	2	2	-	-	-	-
Fluconazole	-	-	-	-	2	2	2	2

Ec: *Escherichia coli* (MTCC-723), Sa: *Staphylococcus aureus* (ATCC-29513), Kp: *Klebsiella pneumonia* (NCTC-13368), Pa: *Pseudomonas aeruginosa* (MTCC-1688), Ao: *Aspergillus oryzae* (MTCC-3567T), An: *Aspergillus niger* (MTCC-281), Af: *Aspergillus flavus* (MTCC-1973), At: *Aspergillus terreus* (MTCC-1782).

**Table 3:** Minimum inhibitory concentration (μg mL^−1^) of the compounds **39a**–**39i**.

Compound	Antitubercular Activity	Antibacterial Activity							Antifungal Activity	
		Gram-Positive Bacteria				Gram-Negative Bacteria				
	Mt	Sa	Se	Ml	Bc	Ec	Pa	Kp	An	Af
**39a**	125.5	62.5	62.5	125	62.5	62.5	125	31.25	62.5	31.25
**39b**	62.5	31.25	31.25	62.5	31.25	62.5	62.5	31.25	31.25	15.62
**39c**	3.9	7.81	1.95	3.9	7.81	7.81	7.81	1.95	3.9	1.95
**39d**	7.81	7.81	3.9	7.81	7.81	7.81	7.81	3.9	7.81	3.9
**39e**	62.5	62.5	31.25	62.5	62.5	62.5	62.5	31.25	62.5	31.25
**39f**	3.9	125	3.9	3.9	7.81	7.81	3.9	1.95	3.9	1.95
**39g**	7.81	15.62	3.9	7.81	7.81	15.62	7.81	3.9	7.81	3.9
**39h**	>125	125	125	>125	>125	125	>125	62.5	62.5	62.5
**39i**	125	125	125	>125	125	62.5	125	62.5	62.5	31.25
Standard	0.97	125	1.95	3.9	7.81	7.81	3.9	1.95	7.81	3.9

Mb: *Mycobacterium tuberculosis*, Sa: *Staphylococcus aureus* ATCC 9144, Se: *Staphylococcus epidermidis* ATCC 155, Ml: *Micrococcus luteus* ATCC 4698, *Bacillus cereus* ATCC 11778, Ec: *Escherichia coli* ATCC 25922, Pa: *Pseudomonas aeruginosa* ATCC 2853, Kp: *Klebsiella pneumoniae* ATCC 11298, An: *Aspergillus niger* ATCC 9029, Af: *Aspergillus fumigatus* ATCC 46645. Standard: Isoniazid: reference standard against *M. tuberculosis*, Ciprofloxacin: standard for other bacteria, Ketoconazol: reference standard for fungi.

**Table 4:** Inhibitory rates of the compounds **55**–**56** against four phytopathogenic fungi at 100 ug mL^−1^.

Compound	*B. cinerea*	*R. solani*	*F. graminearum*	*A. solani*
**55a**	65.53	63.34	39.21	29.71
**55b**	83.11	64.84	52.37	34.57
**55c**	72.37	62.34	51.38	52.57
**55d**	79.68	69.08	52.63	58.28
**55e**	85.62	63.34	45.52	6.00
**55f**	85.39	62.84	47.37	68.28
**56a**	43.38	38.65	21.05	21.42
**56b**	45.66	30.17	18.42	29.14
**56c**	44.98	39.90	22.36	24.57
**56d**	52.28	36.66	25.79	34.00
**56e**	52.28	34.91	17.36	62.57
**56f**	41.55	45.38	25.00	30.85
Hymexazol	100.00	72.82	68.16	55.43

**Table 5:** In vitro antimicrobial activity of benzimidazole–pyrazoles **61a**–**61c**.

Compounds	Microorganisms (μg mL^−1^)							
	*B. subtilis*	*C. tetanis*	*S. pneumoniae*	*E. coli*	*S.typhi*	*V. cholera*	*A. fumigatus*	*C. albicans*
**61a**	100	200	100	200	250	250	>1000	250
**61b**	500	200	200	250	250	50	200	>1000
**61c**	250	250	250	62.5	200	100	>1000	250
Ciprofloxacin	50	100	50	25	25	25	-	-
Chloramphenicol	50	50	50	50	50	50	-	-
Norfloxacin	100	50	10	10	10	10	-	-
Ampicillin	250	250	100	100	100	100	-	-
Griseofulvin	-	-	-	--	-	-	100	500

**Table 6:** Antibacterial activity of the compound **74** and the standards (MIC μg mL^−1^).

Compound	*Staphylococcus aureus*	*Bacillus subtilis*	*Escherichia coli anaerobic*	*Salmonella typhimurium*
**74**	25.3	23.3	16.5	13.4
Chloramphenicol	24.5	32.4	-	-
Cephalothin	-	-	24.3	28.5

**Table 7:** The antimicrobial activity of the compounds **76a**, **76b**, **77b**, **78a**, **78b**.

Microorganism	Inhibition Zone Diameter (mm/mg Sample)					
	76a	76b	77b	78a	78b	Amoxicillin
*Bacillus cereus*	10	7	9	7	10	22
*Bacillus subtilis*	-	-	10	7	9	25
*Staphylococcus aureus*	10	10	18	10	18	16
*Escherichia coli*	7	12	8	8	-	22
*Pseudomonas aeruginosa*	10	9	11	11	13	30
*Salmonella typhimurium*	40	-	-	-	11	20

**Table 8:** The antimicrobial activity of the compounds **80a**–**80h** and **81a**–**81h**.

Compounds	Zone of Inhibition at 100 μg mL^−1^ (in mm)					
	*E. coli*	*P. aeruginosa*	*S. aureus*	*B. subtilis*	*C. albicans*	*A. niger*
**80a**	-	-	-	15	-	-
**80b**	14	13	14	16	-	-
**80d**	12	17	-	-	-	-
**80e**	-	12	12	-	-	-
**80f**	-	-	14	18	-	-
**80g**	13	-	-	-	-	-
**80h**	19	16	18	15	-	-
**81b**	-	-	-	-	13	16
**81c**	-	-	14	13	-	-
**81d**	17	15	-	-	18	-
**81f**	-	-	-	12	14	17
**81g**	-	13	15	-	-	-
**81h**	-	-	-	-	-	14
Gentamycin	22	22	19	20	-	-
Ketoconazole	-	-	-	-	25	20

**Table 9:** Antibacterial and antifungal activities of compounds **83a**–**83b**.

Compounds	MIC, μg mL^−1^ (mM)			
	*B. cereus*	*S. aureus*	*C. albicans*	*A. fumigatus*
**83a**	1250 (5.48)	156.25 (0.684)	625 (2.74)	312.5 (1.37)
**83b**	1250 (4.57)	2500 (9.15)	1250 (4.57)	625 (2.29)
Ampicillin	1250 (3.58)	312.5 (0.894)	-	-
Fluconazole	-	-	2500 (8.16)	-

**Table 10:** Minimum inhibitory concentration in μg mL^−1^ of the compounds **88a**–**88l**.

Compounds	Sa	Se	Ml	Bc	Ec	Pa	Kp	An	Af
**88a**	31.25	31.25	15.62	62.5	31.25	31.25	15.62	62.5	31.25
**88b**	125	62.5	31.25	125	62.5	125	31.25	125	125
**88c**	125	62.5	125	125	62.5	62.5	31.25	125	125
**88d**	62.5	31.25	31.25	31.25	62.5	62.5	31.25	125	62.5
**88e**	15.62	15.62	7.81	15.62	15.62	7.81	7.81	31.25	15.62
**88f**	62.5	31.25	31.25	31.25	62.5	125	31.25	125	62.5
**88g**	15.62	7.81	7.81	7.81	15.62	7.81	7.81	31.25	7.81
**88h**	31.25	15.62	15.62	15.62	15.62	15.62	7.81	31.25	15.62
**88i**	15.62	7.81	3.9	7.81	7.81	15.62	7.81	15.62	7.81
**88j**	31.25	31.25	15.62	31.25	31.25	62.5	15.62	125	31.25
**88k**	62.5	62.5	31.25	31.25	62.5	125	31.25	125	125
**88l**	31.25	15.62	15.62	15.62	31.25	15.62	7.81	31.25	15.62
Ciprofloxacin	15.62	7.81	7.81	7.81	15.62	7.81	3.9	-	-
Ketoconazole	-	-	-	-	-	-	-	15.62	7.81

Sa: *Staphylococcus aureus* ATCC 9144; Se: *Staphylococcus epidermidis* ATCC 155; Ml: *Micrococcus luteus* ATCC 4698; Bc: *Bacillus cereus* ATCC 11778; Ec: *Escherichia coli* ATCC 25922; Pa: *Pseudomonas aeruginosa* ATCC 2853; Kp: *Klebsiella pneumoniae* ATCC 11298, An: *Aspergillus niger* ATCC 9029; Af: *Aspergillus fumigatus* ATCC 46645.

**Table 11:** MIC values and SAR studies in pyrazole series **17a**–**17i**.

Compounds				Minimum Inhibitory Concentration μg/mL				
	C_5_	C_6_	C_7′_	Sa	Sa + HS	E fs	E fm	S p
**98a**	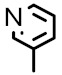	H	H	0.25	4	0.063	-	0.016
**98b**	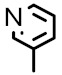	F	H	0.25	2	0.063	-	0.032
**98c**	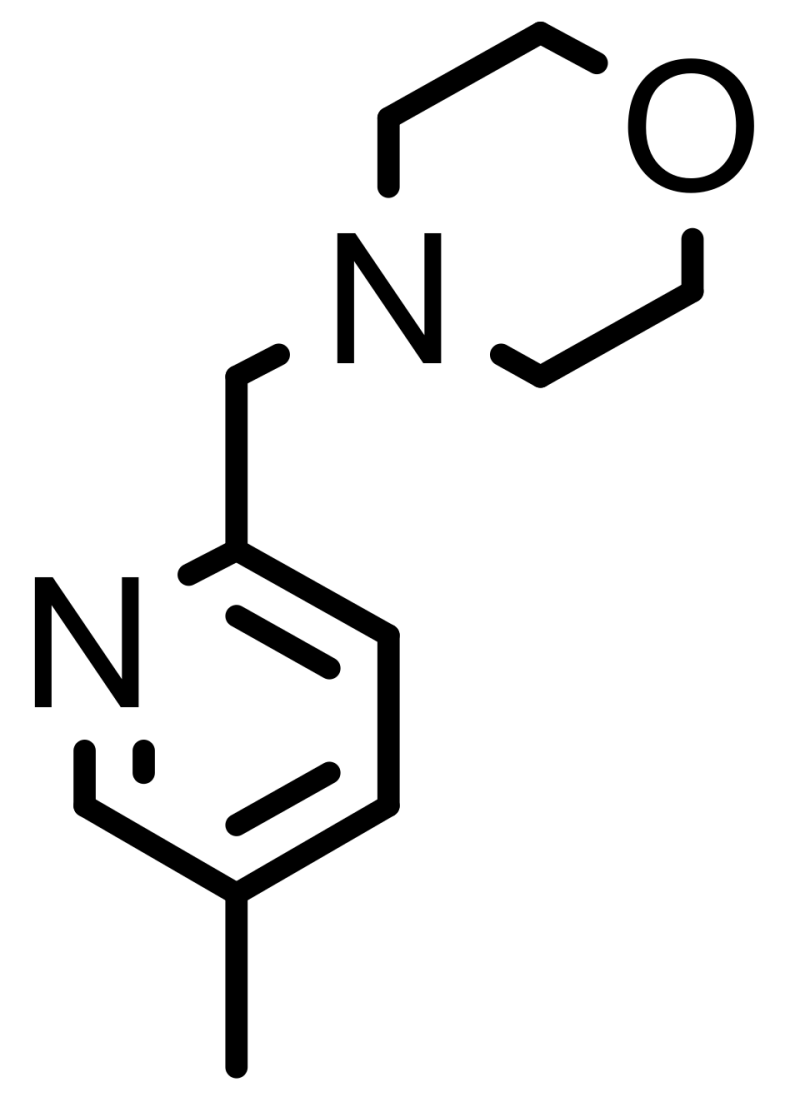	F	H	0.25	1	0.125	0.5	0.016
**98d**	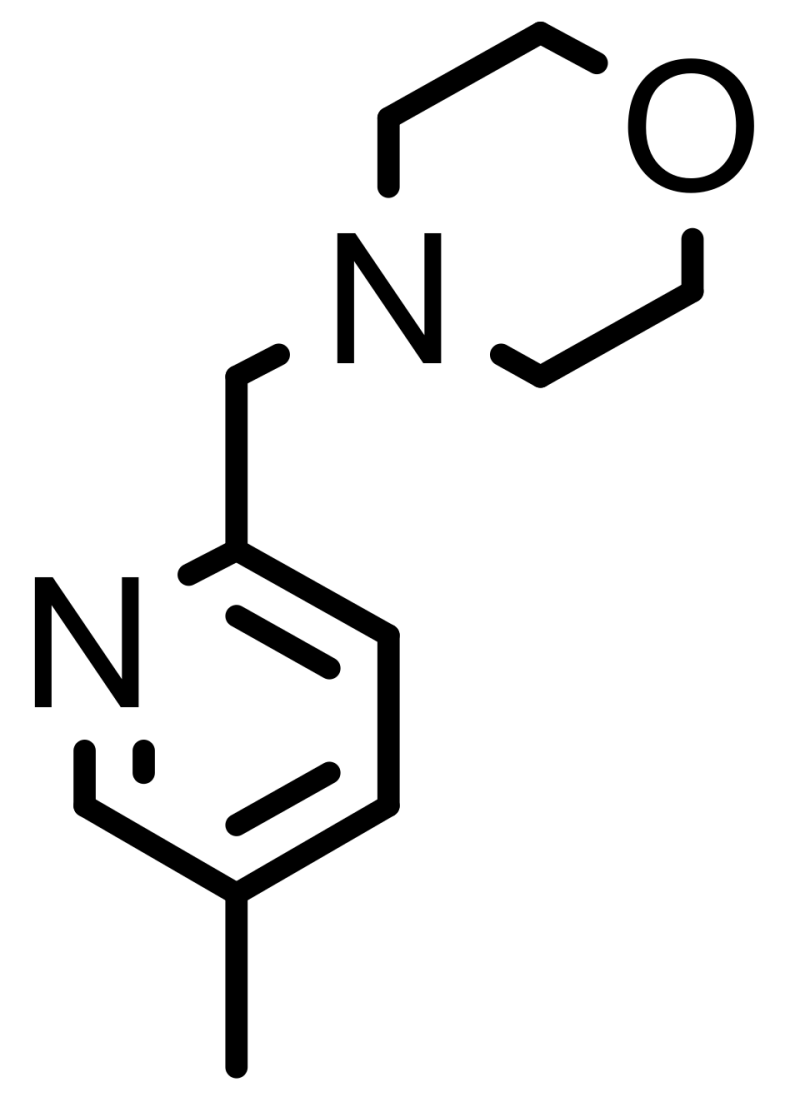	F	Me	0.063	0.5	0.063	0.25	0.016
**98e**	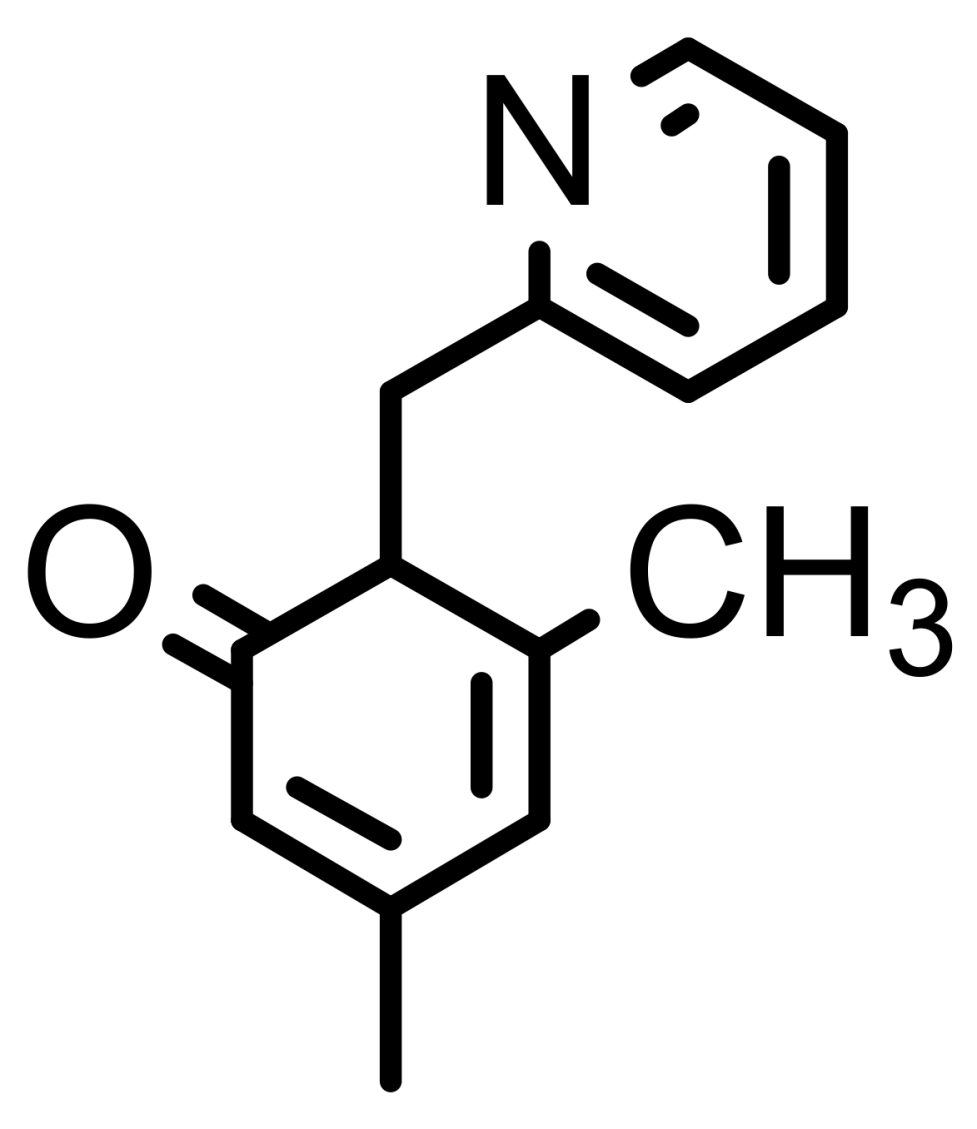	F	Me	0.016	0.125	0.016	0.032	< 0.008
**98f**	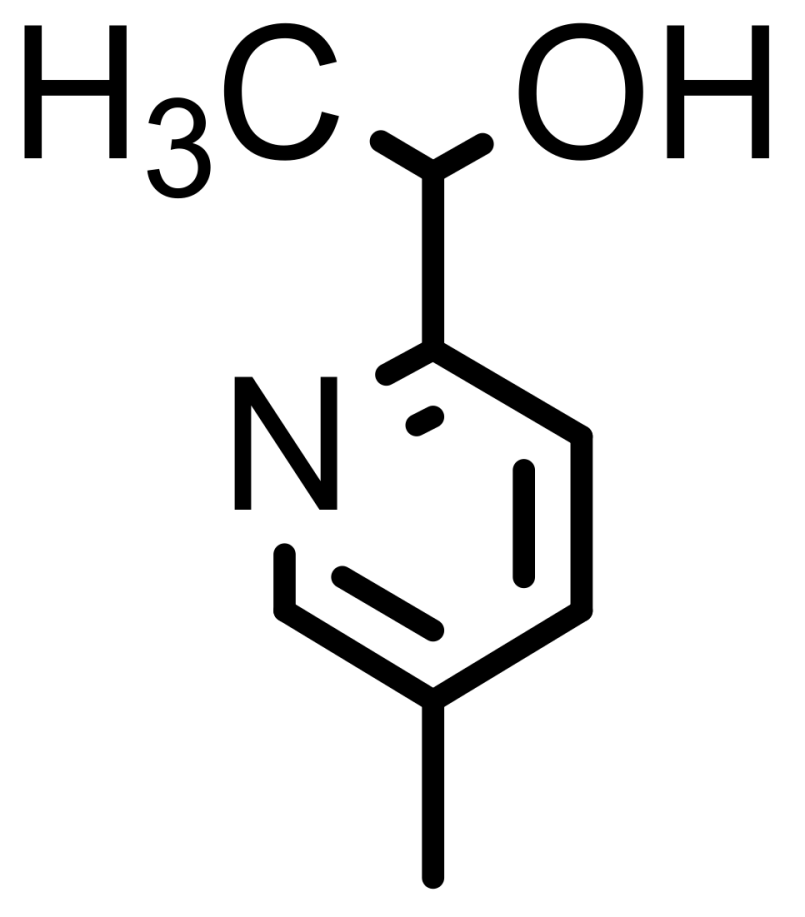	F	H	0.125	3	0.125	0.5	0.032
**98g**	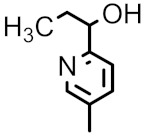	F	H	0.063	2	0.125	0.25	0.032
**98h**	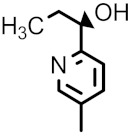	F	H	0.25	4	0.063	0.25	0.016
**98i**	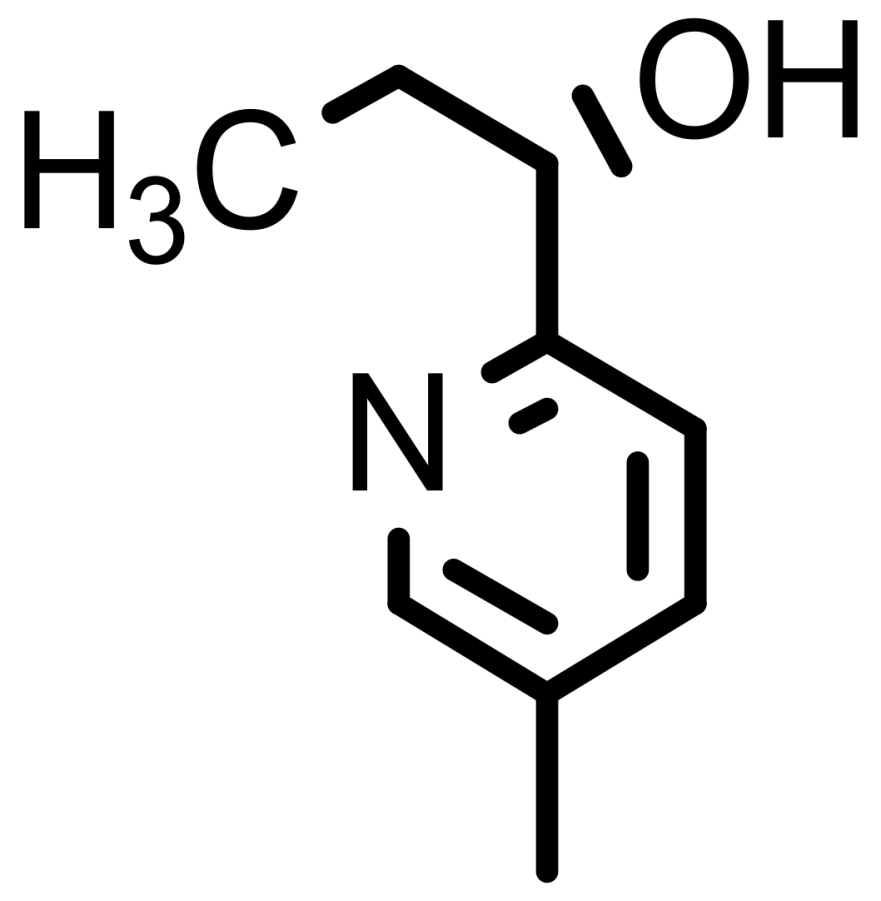	F	H	0.063	1	0.063	0.5	0.063

Sa = *S. aureus*; Sa + HS = *S. aureus* + 50% human serum; E fs = *E. faecalis*; E fm = *E. faecium*; S p = *S. pneumoniae*.

**Table 12:** Gyrase and Topoisomerase IV Inhibition and antibacterial activities of the compounds.

Compound	Enzyme Inhibiton Data Ki (μM)			Minimum Inhibitory Concentration (μg/mL)		
	*S. aureus* gyrase	*E. coli* gyrase	*E. coli* topoIV	*S. aureus*	*S.pneumoniae*	*H. influenzae*
**101**	0.015	<0.004	0.046	0.063	0.008	1
**102**	0.016	<0.004	0.058	0.031	<0.008	0.25

**Table 13:** In vitro antitumor activity of the compounds **83a**–**83b** toward Hep-G2, HCT-116 and MCF-7 cancer cell lines.

Compound	IC_50_ (mM)		
	HepG2	HCT-116	MCF-7
**83a**	0.054	0.042	0.062
**83b**	0.265	0.20	0.281
5-Fluorouracil	0.061	0.041	0.0415

**Table 14:** In vitro antitumor activity of the compounds **109a**–**109d** toward A-549, HCT-116, Hep-G2 and MCF-7 cancer cell lines.

Compounds	% Inhibitory Activity against Tumor Cell Lines at 300 μM			
	A-549	HCT-116	Hep-G2	MCF-7
**109a**	7.2	81.1	40.0	35.1
**109b**	38.3	65.5	60.8	49.4
**109c**	0.0	35.3	41.7	39.5
**109d**	48.8	49.9	62.4	59.6
Doxorubicin	84.1 ± 12.6	111.7 ± 20.5	63.6 ± 9.4	163.8 ± 10.1

## Abbreviations

AcOH	Acetic acid
AcONa	Sodium acetate
BTBA	Benzyltributylammonium chloride
CTAB	etyltrimethylammonium bromide
DCC	N,N′-dicyclohexylcarbodiimide
DMAP	N,N-dimethylpyridin-4-amine
DME	Dimethoxyethane
DMF	Dimethylformamid
DMSO	Dimethylsulfoxid
EDC*HCl	N-(3-Dimethylaminopropyl)-N′-ethylcarbodiimide hydrochloride
Et	ethyl
EtOH	Ethanol
MeOH	Methanol
NBS	N-Bromsuccinimid
PPA	polyphosphoric acid
Ph	phenyl
Py	pyridine
TEA	triethilamine
